# Contrasting environmental conditions precluded lower availability of Antarctic krill affecting breeding chinstrap penguins in the Antarctic Peninsula

**DOI:** 10.1038/s41598-023-32352-7

**Published:** 2023-03-31

**Authors:** Nuria Salmerón, Solenne Belle, Francisco Santa Cruz, Nicolás Alegria, Júlia Victória Grohmann Finger, Denyelle Hennayra Corá, Maria Virginia Petry, Cristina Hernández, César A. Cárdenas, Lucas Krüger

**Affiliations:** 1 International Master of Science in Marine Biological Resources (IMBRSea), Ghent University, Krijgslaan 281/S8, Ghent, Belgium; 2 Departamento Científico, Instituto Antártico Chileno, Plaza Muñoz Gamero, 1055 Punta Arenas, Chile; 3 Instituto de Investigación Pesquera (INPESCA), Colón, 2780 Talcahuano, Chile; 4 Millennium Institute Biodiversity of Antarctic and Subantarctic Ecosystems (BASE), Las Palmeras, 3425 Ñuñoa, Santiago Chile; 5 Laboratório de Ornitologia e Animais Marinhos, Universidade do Vale do Rio dos Sinos (UNISINOS), Av. Unisinos, 950, São Leopoldo, Rio Grande do Sul Brazil; 6 Universidad de Magallanes, Avenida Bulnes, 01855 Punta Arenas, Chile

**Keywords:** Behavioural ecology, Animal behaviour, Marine biology

## Abstract

Dramatic decreases of chinstrap penguin populations across the Antarctic Peninsula (AP) are thought to be influenced by climate-driven changes affecting its main prey, the Antarctic krill, however, empirical evidence supporting such hypotheses are scarce. By coupling data on breeding chinstrap penguins, environmental remote sensing and estimates of krill acoustic density, we were able to demonstrate that penguins substantially increased their foraging effort in a year of low krill availability, with consequent reduction in breeding success. A winter of low sea ice cover followed by a summer/spring with stronger wind and lower marine productivity explained the lower and deeper krill availability. Our results highlight the importance of environmental variability on penguin populations, as variability is expected to increase under climate change, affecting foraging behaviour responses.

## Introduction

One of the key species in the Antarctic food webs that has been affected by climate change is the Antarctic krill, *Euphausia superba*^1^. The increased vertical stratification caused by warming, freshening and sea ice decline, can alter nutrient availability within the surface mixed layer, with a direct impact on primary producers and, as a result, in krill availability^2^. The totality of changes in ocean warming and its consequences will probably influence the population dynamics of several species of krill-dependent predators by modifying behavioural responses^3^. While there is some variability across the Southern Ocean, krill recruitment and abundance depends on suitable habitat conditions usually influenced by sea-ice dynamics^4,5^. Low sea-ice coverage in winter also reduces food availability for krill larvae in the spring and summer, as sea ice melting releases nutrients responsible for algal blooms^6,7^. According to some studies, Antarctic krill stocks have declined between 38–81% between 1976 and 2003 in the Southwest Atlantic sector of the Southern Ocean with an alleged distribution displacement towards the Antarctic shelves^8^.

The chinstrap penguin (*Pygoscelis antarcticus*) is an abundant species in the Antarctic Peninsula that, during breeding, feeds almost exclusively on Antarctic krill^9,10^. Studies have highlighted significant declines on chinstrap populations^11,12^. A study^13^ proposed a holistic hypothesis for this decline, linking chinstrap penguin population with krill biomass. The main factors accepted to affect krill populations that could have cascading effects in chinstrap penguin abundance are the changes on sea ice cover caused by climate change^4,5,14^, the increased competition for krill caused by the recovery of whale populations and the growth of krill-trawling fisheries^15–18^. A recent publication^19^ also showed that the joint effects of sea ice, storms and cloudy conditions affect the phenology of low and mid trophic levels of marine ecosystems in the Antarctic Peninsula, therefore having a bottom-up effect over top-predators, particularly, causing a mismatch between the peak food availability and penguins’ breeding.

Chinstrap penguins are central place foragers during breeding, therefore breeders are restricted to continuously returning to the nest after a foraging trip to attend incubation or feed the offspring. Hence, their foraging efficiency during breeding is dependent upon the ability to find food within the range of accessible habitat^20^. The critical months for chinstrap penguin breeding are typically December to February^21^, when energetic demands from hatching and growing chicks increase. If the duration of provisioning trips exceeds a critical threshold, or birds are unable to capture sufficient prey within a given time, reduced breeding success can occur^22^. This can have a strong effect on chicks survival and population size in the long run. Also, a temporal uncoupling or alteration in the ability of adults to match the start of the chick-rearing feeding period and the maximum availability of food, can generate failures in reproductive success^23^. In this regard, current and projected climate changes can play a key role, in fact, some studies have already provided evidence on how environmental variability (e.g. wind-driven downwelling) can influence penguin foraging behaviours that are indicators of changes in krill availability^24^.

Understanding of ecological mechanisms underpinning chinstrap penguin behaviour and population dynamics in response to fluctuations in krill availability is crucial to develop management and conservation strategies under a scenario of higher environmental variability and increased krill fishing in the Antarctic Peninsula AP^25–27^. Hereby, the aims of this study are to quantify and compare foraging metrics of chinstrap penguins from a population in the tip of the AP during two breeding seasons between 2019 and 2022, and investigate the links between foraging behaviour differences and environmental variability. We hypothesised that winter sea ice conditions and stronger winds affecting spring/summer productivity reduce krill availability, causing a cascade effect on Chinstrap Penguin foraging behaviour and population-level breeding success.

## Materials and methods

### Study area

**Figure 1:**
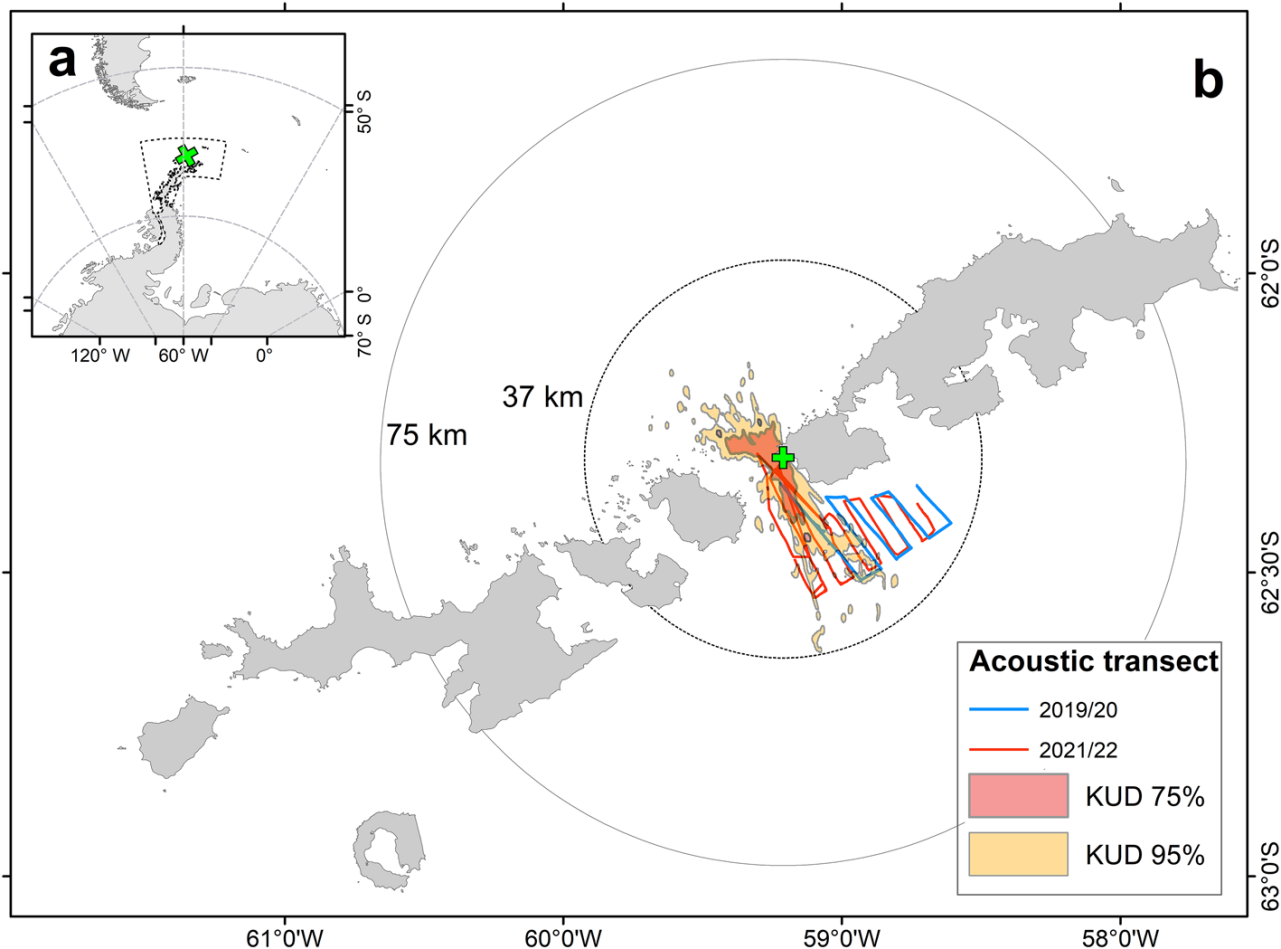
Location of the study area over a wide (**a** subarea 48.1, dashed line) and over a local geographical context (**b**). The green cross shows the position of Harmony Point, Nelson Island, where the foraging range (Kernel Usage Densities KUD) and foraging behaviour of breeding chinstrap penguins were studied in 2019/20 and 2021/22 breeding seasons. A 37 km radius around the penguin breeding colony indicates the farthest distance from the colony on a straight line; environmental variables compared between seasons were extracted within a 75 km radius around the breeding colony. Solid lines depicted the acoustic transects carried out on board the RS Karpuj in both seasons. Map created using ArcMap 10.8.2 (https://www.esri.com/). Land contours from Esri Global Mapping International (https://www.arcgis.com/; item id a3cb207855b348a297ab85261743351d) and the SCAR Antarctic Digital Database [2022] (https://www.add.scar.org/). Subarea 48.1 from FAO Fisheries GeoNetwork Platform (https://www.fao.org/fishery/geonetwork/).

The study was undertaken during the 2019/20 and 2021/22 austral summer season (December–February) at Harmony Point, Nelson Island, South Shetland islands (SSI), in the maritime Antarctic Peninsula AP (Fig. 1a,b). This area is recognized as an Antarctic Specially Protected Area (ASPA No. 133) due to its rich biota, especially birds, and one of the larger chinstrap colonies in the South Shetland Islands*.*

### Penguin foraging trips and dives

Chinstrap penguins were captured by hand on the nest during duty shifts in late December and early January, corresponding to late incubation and early chick-rearing. The individual about to leave the nest for foraging was captured when its pair arrived at the nest, therefore nests were always guarded by one adult. Each captured animal had its head covered by a hood to reduce stress^28^. Twenty seven breeding penguins were instrumented (17 in 2019/20 and 10 in 2021/22), totalling 130 foraging trips. Axy-trek marine loggers (40 × 20 × 8 mm, 14 g, GPS logger, time depth recorder TDR and accelerometer) were attached to the dorsal feathers using 3 M Extreme Hold Duct Tape 2835-B (1.88 inches) and Loctite super glue. GPSs were programmed to record a location every 5 min and TDRs recorded depth every 1 s. Instruments were deployed for 5–10 days, after which the animal was recaptured and the device removed. Animal handling during deployment took less than 10 min and between 6 and 8 min on recovery, after which all individuals were returned immediately to their nests. During recovery one researcher would watch the eggs or chicks for predators until the adult was released back to the nest.

GPS fixes were interpolated to correct for interruptions and standardise fixes every 5 min in the ‘adehabitatLT’ R package^29^ and treated with a speed filter set to 10 m/s to remove unreal velocity outliers. Fixes positioned < 250 m from the breeding colony were removed. A time filter was used, so only trips > 1 h were used in the analysis. Dives were processed using the R package ‘diveMOVE’^30^. The metrics calculated were foraging trip duration (h), cumulative trip distance (the total distance in km covered by the animal during the trip), frequency of complete dives (from all dives, the proportion of dives with bottom phase indicating prey was found, Fig. S1), mean dive duration (minutes), cumulative dive duration (total time spent in diving), mean and maximum depth during the bottom phase of the dive (m), and mean capture effort, calculated as the number of wiggles per dive^31^. Wiggles are deviations of the depth during the bottom phase, which are potential indicators of feeding events^32,33^. See Appendix S1 Fig. S1 for examples of wiggles.

### Breeding success

A drone DJI Mavic 2 Pro with Hasselblad camera was flown over the sampled breeding sub-colony and neighbour sub-colonies during mid incubation (December, each season) and during creché (February, each season) to take images at 80 m elevation. Imagery was used to construct a georeferenced orthomosaic in order to count nests and chicks for the sub-colonies. The number of chicks raised per nest was calculated for each sub-colony by dividing the number of chicks in crèche by the number of nests during incubation.

Parameters were compared between the two seasons using Permutational Analysis of Variance (PERMANOVA) in the ‘PERMANOVA’ R package^34^ with 999 permutations. All variables were standardised by removing the means and dividing by the standard deviation.

### Ethics and environmental approval

Methods for working with live animals were approved by the Magallanes University ethical committee (*Comité de Ética Científico de Universidad de Magallanes*) in the certification number 069/CEC/2018 and by the *Comité de Bioética, Seguridad, Cuidado y Uso de Animales de la Fundación Instituto de Biodiversidad de Ecosistemas Antárticos y Subantárticos* (BASE) in the certification number 3/CBSCUA/2022; environmental permits issued by Instituto Antártico Chileno INACH numbers 1045/2019, 1046/2019, 654/2021, 661/2021 and 433/2022, which authorise sampling and entrance in protected areas also taking into account the previous ethical approval by the Magallanes University ethical committee. Instituto Antártico Chileno INACH, as one of the institutions representing Chile in the Antarctic Treaty, has a specific department responsible for evaluating environmental impact from research and issuing environmental permits for the execution of research in Antarctica by all Chilean institutions.

### Ethics and environmental guidelines

We follow the guidelines in the Magallanes University ethical committee certification number 069/CEC/2018, in the *Comité de Bioética, Seguridad, Cuidado y Uso de Animales de la Fundación Instituto de Biodiversidad de Ecosistemas Antárticos y Subantárticos* certification number 3/CBSCUA/2022 and INACH permits No 1045/2019, 1046/2019, 654/2021, 661/2021 and 433/2022. Reporting of methods and results involving live animals adhere to the ARRIVE guidelines^35^.

### Krill acoustic abundance

Acoustic surveys were carried out in the research vessel “*RS Karpuj*”. Each survey followed a 500 km (linearly) transect covering the foraging area of penguins (Fig. 1). Acoustic data was recorded using a multi-frequency scientific echo sounder, SIMRAD EK60, with split beam transducers (38, 120 and 200 kHz) calibrated with metal spheres (cooper in 2019/20 and tungsten in 2021/22)^36,37^. Pulse length frequency was set at 1.024 ms for all frequencies and the ship's speed was maintained at approximately ten knots. The data collection range was set from 0 to 500 m deep, and was recorded only during daylight hours.

Acoustic data was analysed with the Echoview post processing software version 9.1. Acoustic data was filtered by eliminating electrical interference, noise of mechanical origin, pings attenuated by bubbles, double-bottom echoes and a buffer near the transducer, to avoid the ringdown effect. After filtering the data, we proceeded to identify and select the swarms of Antarctic krill following the Commission for the Conservation of Antarctic Marine Living Resources (CCAMLR) methodology for the identification and integration of krill swarms recommended in SG-ASAM-17 (Appendix S1 Fig. S2). The acoustic information was echo-integrated into basic sampling units of 0.5 nautical miles by 5 m deep and converted into NASC (Nautical Area Scattering Coefficient, m^2^ nmi^−2^). NASC measurements have been used as a proxy of potential prey distribution and availability in the water column^38–40^.

Three krill abundance parameters were compared between seasons: (1) total acoustic density (NASC); (2) swarms’ depth range (m), where depth for the swarms with density higher than the 1st quartile (> 10 mean NASC) were used, and (3) swarms’ aggregation based on Ripley´s K index^41^: first, only horizontal data with NASC detection was selected, and isotropic corrected K_iso_ was calculated for each vertical 5-m strata. The K_iso_ was subtracted from the expected K; higher values in relation to the expected indicate increased krill aggregation (patchier), in contrast, lower values in relation to the expected indicate that krill is more dispersed (Appendix S1 Fig. S3). Krill parameters were compared between seasons (2019/20 and 2021/22) using PERMANOVA based on 999 permutations and standardisation as previously.

### Local conditions for penguins

In order to evaluate whether the environmental conditions differed between 2019/20 and 2021/22, time averaged maps of chlorophyll-a concentration CHL^42^, photosynthetically available/active radiation PAR^43^ and surface wind speed SWS^44^ between November and January, and fractional sea ice cover SIC^44^ between June and Septemberof the immediately previous winter (wSIC from now on) were downloaded from the Giovanni National Aeronautics and Space Administration (NASA) browser (giovanni.gsfc.nasa.gov). Those variables were extracted into a 5 km × 5 km spatial grid within a 75 km radius around the breeding colony (Fig. 1). Variables were compared between seasons using a PERMANOVA with 999 permutations and standardisation as previously.

### Time-series analyses of environmental variables

For a global perspective of climate conditions at the whole area 48.1 (Fig. 1), time-series of environmental data were downloaded from the Giovanni NASA browser (see previous section). Monthly averaged data on CHL^42^ and SIC^44^ were downloaded between May 2005 to March 2022 over a spatial extent between 70°W, 70°S and 55°W, 60°S, therefore encompassing the northern part of the Antarctic Peninsula. Means ± sd were calculated for each month over the 18 years to describe longer term trends.

All statistical analyses were conducted in R 4.2.0^45^ (R Core Team 2022). For more details on the analysis, see R codes in Appendix S2.

## Results

### Penguin foraging parameters

Chinstrap penguins foraged within a maximum interval of 24 h, covering ca. 70 km each trip, reaching a maximum of 35 km from the colony on a straight line (Fig. 1). Most dives lasted less than 2 min, and cumulatively they could reach over two hours of dives in a single trip. Dive depths were usually around 40 m and reached a maximum of 133 m. Animals would wiggle between 2 and 17 times per complete dive.

**Figure 2:**
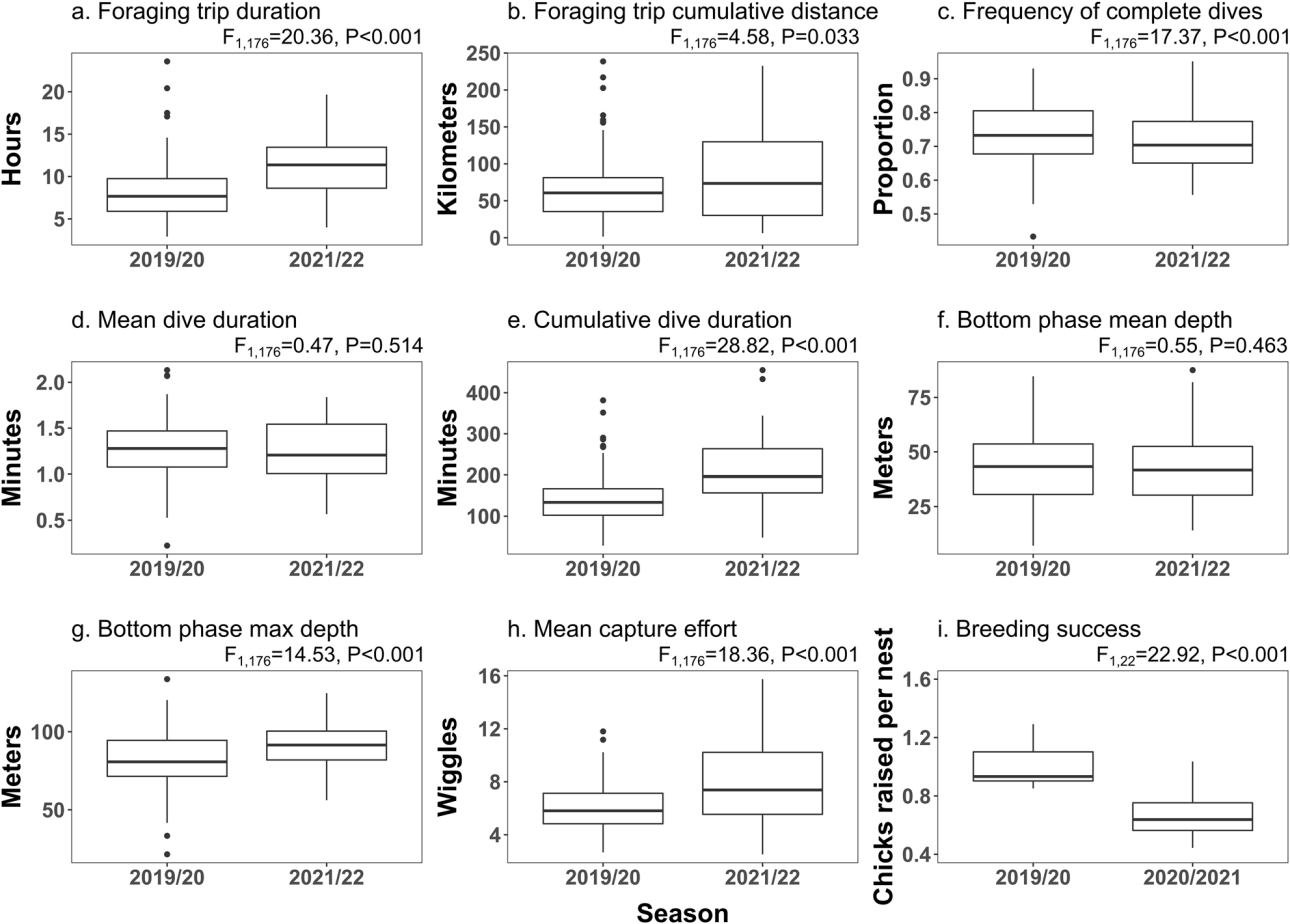
Foraging trips (**a**,**b**), dives (**c**–**h**) and breeding success (**i**) for chinstrap penguins breeding at Harmony Point (Nelson Island) in two seasons with contrasting conditions (2019/20 and 2021/22). Statistical results from Permutational Analysis of Variance (999 permutations).

Foraging trips were longer in 2021/22, both in duration (F_1,176_ = 20.36, *p* < 0.001) and distance (F_1,176_ = 4.58, *p* < 0.033, Fig. 2 a,b). There were less complete dives in 2021/22 compared to 2019/20 (F_1,176_ = 17.37, *p* < 0.001, Fig. 2c), and while the mean dive duration per trip did not differ (F_1,176_ = 0.47, *p* = 0.514, Fig. 2d) the cumulative dive duration was higher in 2021/22 than in 2019/20 (F_1,176_ = 28.82, *p* < 0.001, Fig. 2e). Similarly, no differences were found for mean dive depth (F_1,176_ = 0.55, *p* = 0.463, Fig. 2f) but maximum dive depth was deeper in 2021/22 compared to 2019/20 (F_1,176_ = 14.53, *p* < 0.001, Fig. 2g). Capture effort was also higher in 2021/22 compared to 2019/20 (F_1,176_ = 18.36, *p* < 0.001, Fig. 2h). Breeding success in 2019/20 was 1.4 times higher than in 2020/2021 (F_1,22_ = 22.92, *p* < 0.001, Fig. 2i).

### Krill acoustic abundance

**Figure 3:**
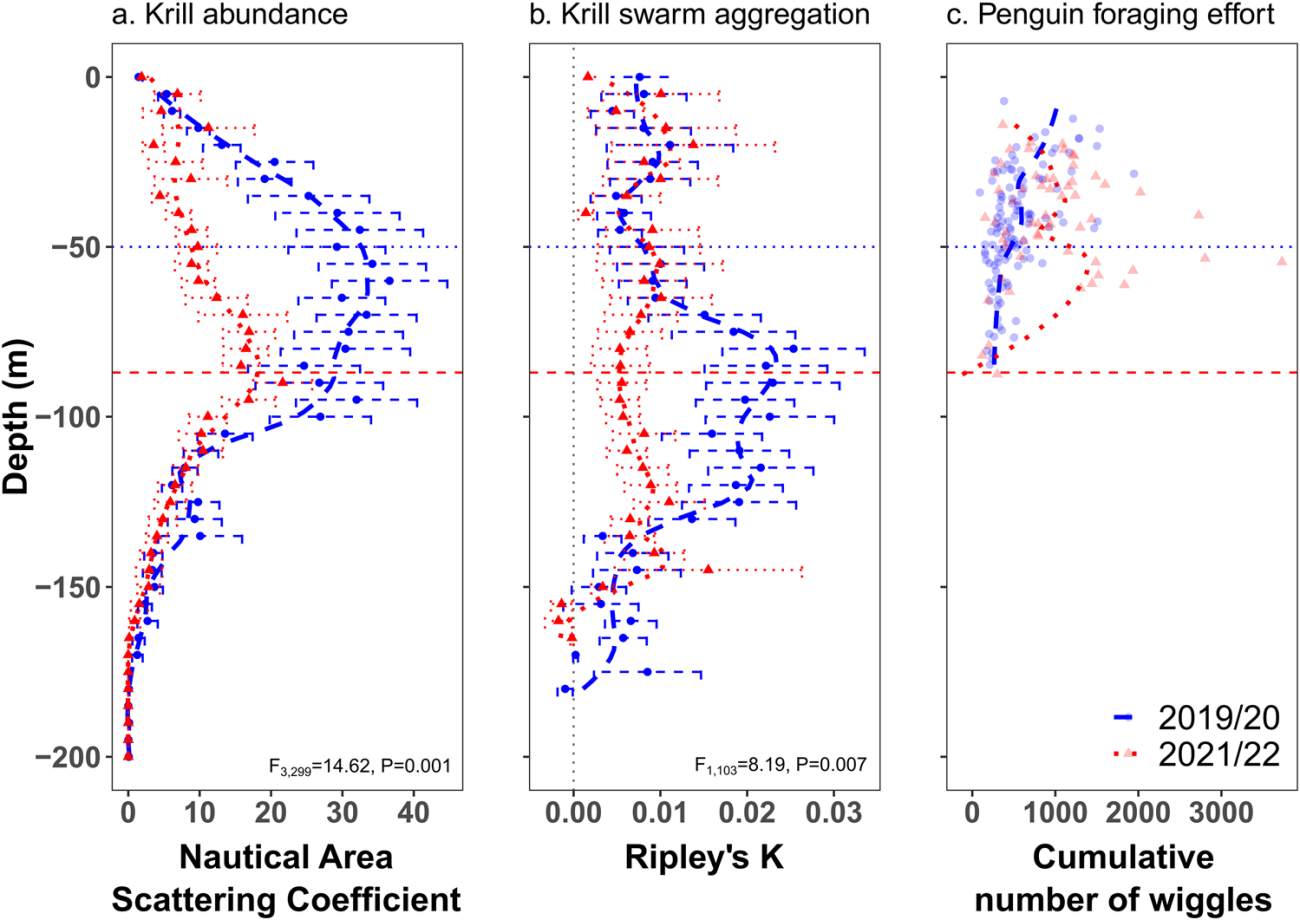
Mean ± s.e. vertical distribution of Antarctic krill (*Euphausia superba*) acoustic density(**a**), Mean ± s.d. Ripley’s K coefficients for measuring krill swarms vertical aggregation (**b**) and vertical foraging effort of chinstrap penguins (**c**) at Harmony Point, Nelson Island, in two seasons with contrasting environmental conditions. Horizontal lines indicate the depths of krill biomass peaks in 2019/20 (blue dotted line) and 2021/22 (red dashed line). Vertical grey dotted line indicates the biomass threshold for comparing depths in both seasons (**a**) and 0 Ripley´s K (**b**). Ripley’s K (**b**) positive values indicate aggregation of swarms, values equal to zero indicate that swarms were randomly distributed, and negative values indicate dispersed or patchier swarms. Bottom phase mean dive depth of each foraging trip and the total number of wiggles per foraging trip (**c**). Statistical results from Permutational Analysis of Variance (999 permutations).

During 2019/20, mean NASC biomass was higher than in 2021/22 (F3,299 = 9.07, *p* = 0.002) and swarms’ depth was lower (F3,299 = 14.62, *p* = 0.001) compared to 2021/22 (Fig. 3a). Overall, swarms tended to aggregate (positive Ripley’s K), but were more aggregated in 2019/20 (F_1,103_ = 8.19, *p* = 0.007). These differences were only evident in depths deeper than 60 m (Fig. 3b), which were deeper than usual diving depths of penguins (Fig. 3c). Vertical distribution of krill in those different seasons (Fig. 3a,b) matched differences in the vertical distribution of penguin foraging effort (Fig. 3c), as in 2019/20 higher effort was focused over shallower depths compared to 2021/22.

### Environmental variability

**Figure 4:**
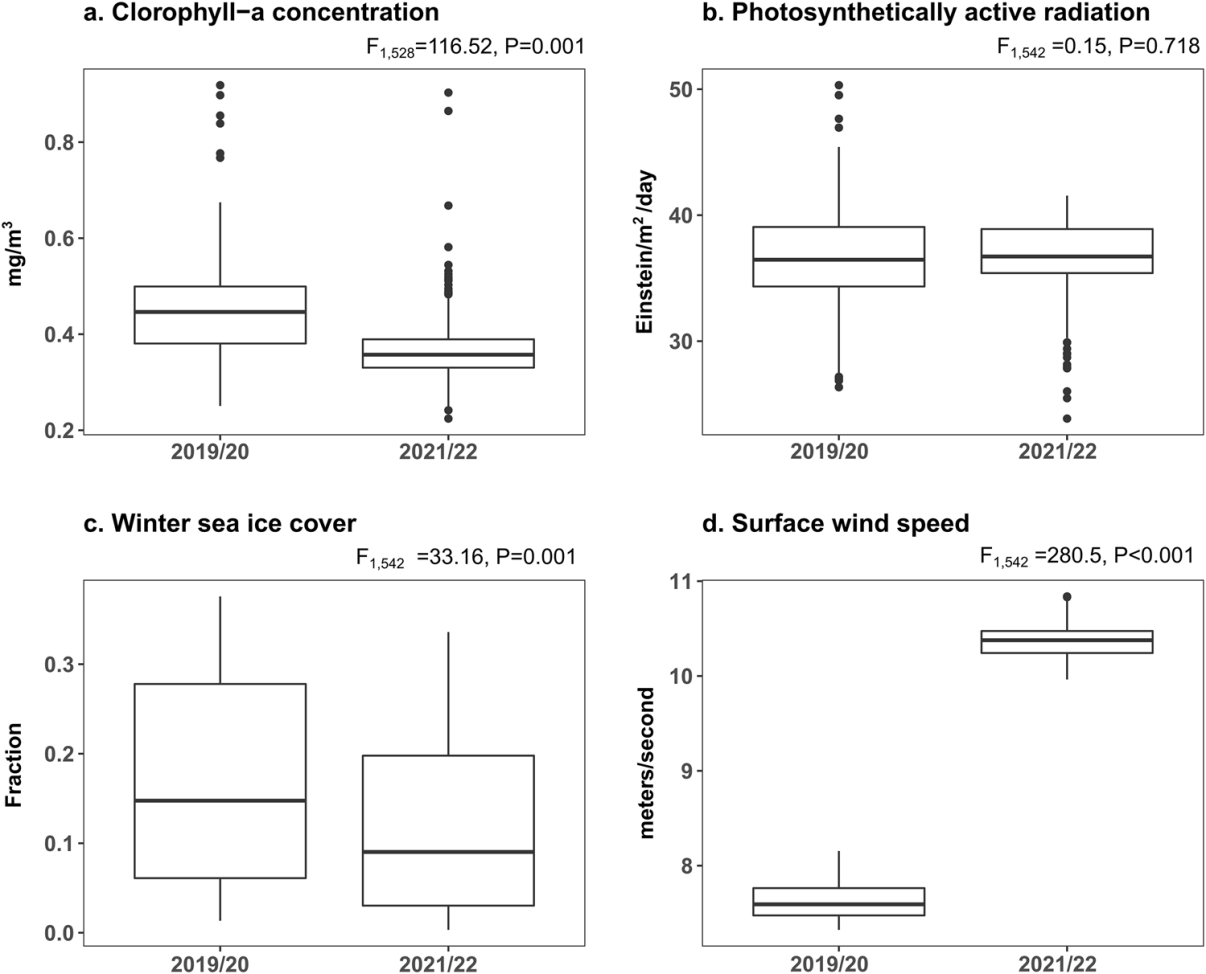
Seasonal differences of spring–summer chlorophyll-a concentration (**a**), spring–summer photosynthetically active radiation (**b**), winter sea ice cover (**c**) and spring–summer surface wind speed (**d**) in a 75 km radius around Harmony Point, Nelson Island in two different seasons. Statistical results from Permutational Analysis of Variance (999 permutations).

Comparing environmental variables between seasons in a 75 km radius around Harmony Point (local scale), CHL concentration was lower in 2021/22 (F_1,528_ = 116.52, *p* = 0.001, Fig. 4a), while PAR was the same between years (F_1,542_ = 0.15, *p* = 0.718, Fig. 4b) and wSIC was lower in 2021/22 (F_1,542_ = 33.162, *p* = 0.001, Fig. 4c). Wind was higher in 2021/22 (F_1,542_ = 280.50, *p* < 0.001, Fig. 4d). We detected an CHL peak that occurred one month earlier in 2021/22 (November) compared to the previous year (Appendix S1 Fig. S4) and two months compared the longer term mean (Appendix S1 Fig. S5). However the evidence to support this is not available due to the monthly resolution of the data (Appendix S1 Fig. S4).

## Discussion

Results from this study highlight the importance of environmental variability on penguin populations. Our results showed that chinstrap penguins adjusted their foraging behaviour to low krill availability conditions by performing longer trips with deeper and more frequent dives, with likely consequences on breeding success. Increased foraging effort is a common response of central place foragers to decreased food availability^46–48^. Most studies that have evaluated changes in effort explained by changes in food availability have used indirect measures of food availability such as indexes of large scale climate systems^46,49^, productivity or vertical position of the mixed layer^24,47^ and the behaviour of the bird itself^31,50^. Studies that used direct indicators of food availability, such as ours, found that effort was higher when food availability was lower, be it in Antarctica^51,52^ or elsewhere^53,54^.

Seabirds usually time their breeding with the high availability of resources^23^; hence, it is expected that the breeding period of penguins is aligned with environmental timing of krill production so that the chick-rearing occurs when food is most available in the vicinity of the colonies and accessible to penguins in the vertical strata^22,55^. Any alteration of this spatio-temporal synchronisation might force adults to increase foraging effort and consequently may impact the food provisioning to chicks, with consequences in breeding success^51,52^. In addition, we detected an anticipation of chlorophyll-a peak in 2021/22, which may have caused a temporal mismatch between maximum food availability and chick-rearing feeding. While that earlier peak is consistent with our hypothesis, we can not prove its importance due a lack of data.

Variability in abundance and distribution of prey are usually linked to local or large-scale oceanographic and climatic processes^51,53,54^. For Antarctic krill, low sea ice cover during winter, an especially critical period of its life cycle, causes a mismatch between iron-dependent phytoplankton blooms and Antarctic krill larvae that can reduce krill productivity^7,56^. The cascade effect of the decline in krill availability over chinstrap penguin foraging behaviour and breeding success could be explained by a joint effect of reduced biomass and deepening of the krill abundance.

Krill tends to show a patchier distribution under low abundance^57^, therefore the distance between krill patches increases and could explain the larger distance covered by chinstrap penguins to increase the probability of finding food sources. At least in our results, a patchier distribution of krill did not seem to affect penguins. Our results also indicate that the reduced productivity due to lower winter sea ice^58^ and the likely deepening of the mixed layer resulting from stronger winds^59^ reduced krill biomass and caused krill to use deeper strata in the water column (this study), hence, there was less krill available for penguins in both horizontal and vertical layers. A previous study in the South Orkney Islands^24^ has found a similar result: stronger winds resulting from a strong but short El Niño event caused a deepening of the upwelling, which, in turn, was followed by an increase in chinstrap penguins’ foraging effort. The observed change in foraging effort was attributed to horizontal and vertical changes in krill availability.

Chinstrap penguins are highly specialised krill foragers^60^; chinstrap populations breeding at the South Shetland Islands, rely almost entirely on krill^9,10^ contrasting to gentoo penguins (*Pygoscelis papua*) whose flexibility allows for shifting diet in periods of low krill availability^61,62^. Not surprisingly, while populations of chinstrap penguins experience a generalised decrease^12^, gentoo penguin populations are increasing on the AP^63^. Our results provide evidence that support early views^13^ that the increasing frequency of low krill biomass events is causing a reduction in penguin breeding success and, hence, population size. The mechanism explained here for the chinstrap decline was based on local conditions observed around Nelson Island colonies. The South Shetland Islands have particular geographic and bathymetric features that shape water flow, krill flux, retention and availability to penguins^64^ and may be distinct from other areas. However, many of the environmental variables included in our study are experiencing similar trends at local and wider scales, making this mechanism plausible as an ecological process propagated among multiple colonies across the AP. Further replication of our approach on other colonies is needed, by synchronously monitoring reproductive success, acoustic krill abundance and environmental variability between seasons, in order to provide support to the hypothesis planted above.

When coupled to fisheries, climatic events can have even greater local effects on krill availability^13,26^. Hypothesis stated that a year of high fishing catches followed by a warm winter with lower concentrations of sea ice could affect penguin breeding populations by reducing krill availability below what penguin populations require^25^. Our results demonstrated that lower winter sea ice conditions had consequences for krill availability in summer. Although we did not measure the effects of fishery, in a season when environmental conditions are not favourable, such as 2021/22, high levels of fishing could affect the krill population itself^18^ and, therefore, lead to punctual effects over penguin populations^26,65^ that have the potential to persist through the following seasons^25^. Of particular concern are the chinstrap penguins whose bulk of the population is located in the AP^12^ and whose breeding success (therefore recruitment in the long run) can be directly related to changes in krill distribution and abundance as a direct effect of warming (this study). If events of low winter sea ice follow recent trends^66^ and krill biomass keeps declining or moving southwards^8,67^ in the AP, foraging behaviour responses like the one observed in this study are predicted to occur more often over the next century for Antarctic krill predators. That should motivate krill fishing managers to adopt more precautionary management strategies, particularly in years (fishing seasons) when krill availability is reduced.

In terms of spatial management, there are Marine Protected Areas (MPAs) that have been proposed with several being under discussion^68^. In the AP, the proposal for the Domain1 MPA (CCAMLR-41/34, SC-CCAMLR-38/BG/03) is still under discussion in CCAMLR, but so far there is no consensus for its adoption. The increase of environmental variability in the AP along with several other physical and anthropogenic drivers are expected for the next decades^3^. Strong changes in krill availability produced by environmental perturbation, potential relocation of both krill and predators and the fishery would also be expected, which makes comprehensive ecosystem scale measures, such as Domain 1 MPA, necessary.

## Supplementary Information

Supplementary Information 1.

Supplementary Information 2.

## Data Availability

The datasets generated and/or analysed during this study can be found online^69,70^ in https://zenodo.org/record/6779360 and https://zenodo.org/record/7044788.
