# Supplementary material for: Contrasting environmental conditions precluded lower availability of Antarctic krill affecting breeding chinstrap penguins in the Antarctic Peninsula
Source: Sci Rep. 2023 Mar 31;13:5265. doi: 10.1038/s41598-023-32352-7 (PMC10066220; doi:10.1038/s41598-023-32352-7)
Supplement: Supplementary file 1 — Supplementary Information 1. [file 41598_2023_32352_MOESM1_ESM.docx]

Supplementary Information

Title: **Contrasting environmental conditions precluded lower availability of Antarctic krill affecting breeding chinstrap penguins in the Antarctic Peninsula**

Nuria Salmerón^1^, Solenne Belle^1^, Francisco Santa Cruz^2^, Nicolás Alegria^3^, Júlia Victória Grohmann Finger^5^, Denyelle Hennayra Corá^5^, Maria Virginia Petry^5^, Cristina Hernández^6^, César A. Cárdenas^2,4^, Lucas Krüger^2,4^*

^1^International Master of Science in Marine Biological Resources (IMBRSea), Ghent University, Krijgslaan 281/S8, Ghent, Belgium.

^2^ Departamento Científico, Instituto Antártico Chileno, Plaza Muñoz Gamero, 1055, Punta Arenas, Chile

^3^ Instituto de Investigación Pesquera (INPESCA), Colón 2780, Talcahuano, Chile

^4^ Millennium Institute Biodiversity of Antarctic and Subantarctic Ecosystems (BASE), Las Palmeras 3425 Ñuñoa, Santiago, Chile

^5^ Laboratório de Ornitologia e Animais Marinhos, Universidade do Vale do Rio dos Sinos (UNISINOS), Av. Unisinos, 950, São Leopoldo, Rio Grande do Sul, Brazil

^6^ Universidad de Magallanes, Avenida Bulnes 01855, Punta Arenas, Chile

*corresponding author: [lkruger@inach.cl](mailto:lkruger@inach.cl) **+56 612 298 100**


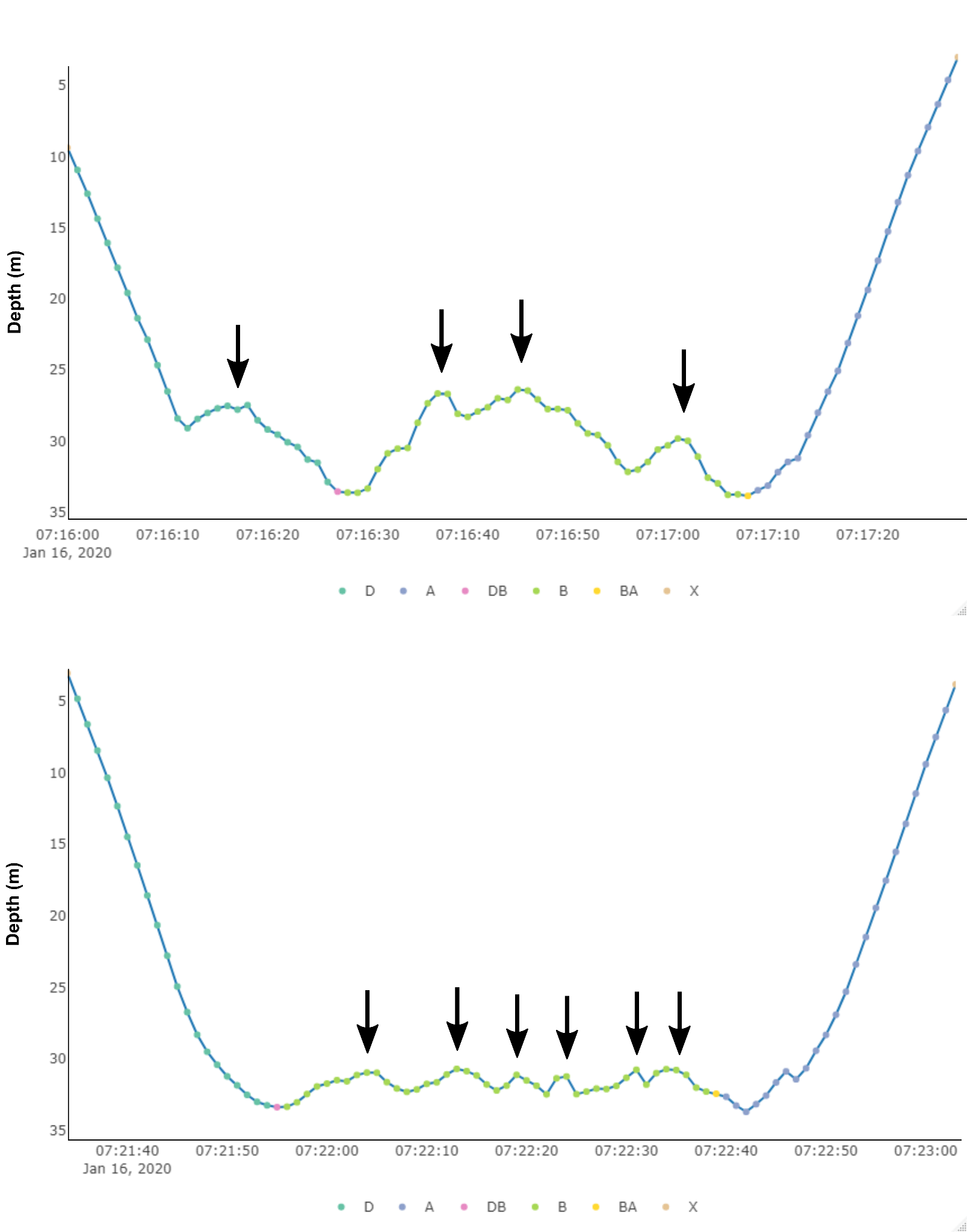


Figure S1. Examples of two complete dive profiles from a single chinstrap penguin breeding at Harmony Point, Nelson Island (maritime Antarctic Peninsula) in January 16, 2020. Different dive phases: descent (D), ascent (A) descent-bottom (DB), bottom (B), bottom ascent (BA), surface (X) and wiggles - a measure of capture effort - indicated by the arrows.


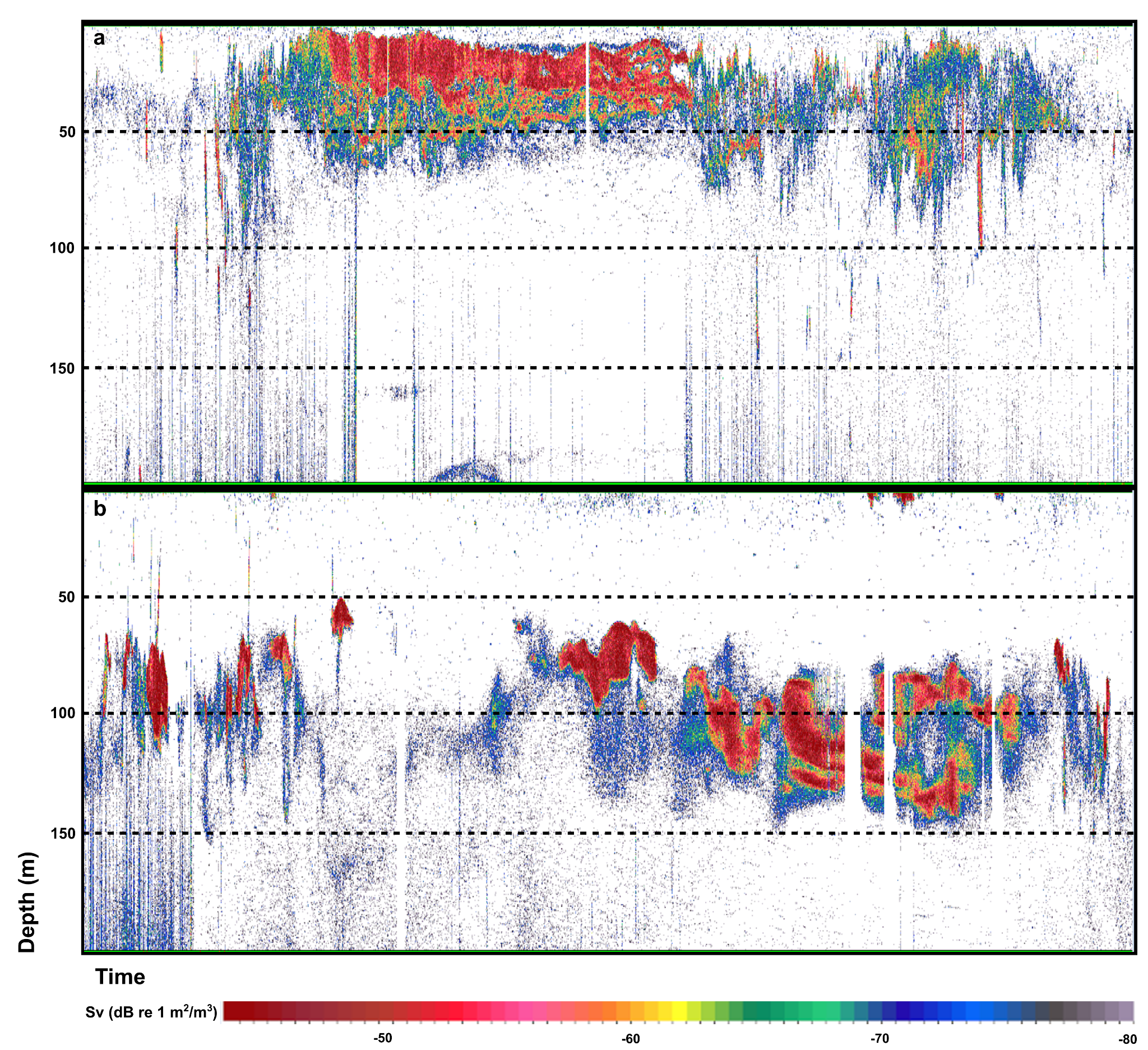


Figure S2. Two examples of 120 kHz echograms showing krill swarms detected by volume backscattering strength (Sv) reflectance in 27 december 2019 (a) and 29 December 2019 (b) within a 30 km radius around Harmony Point, Nelson Island (maritime Antarctic Peninsula).


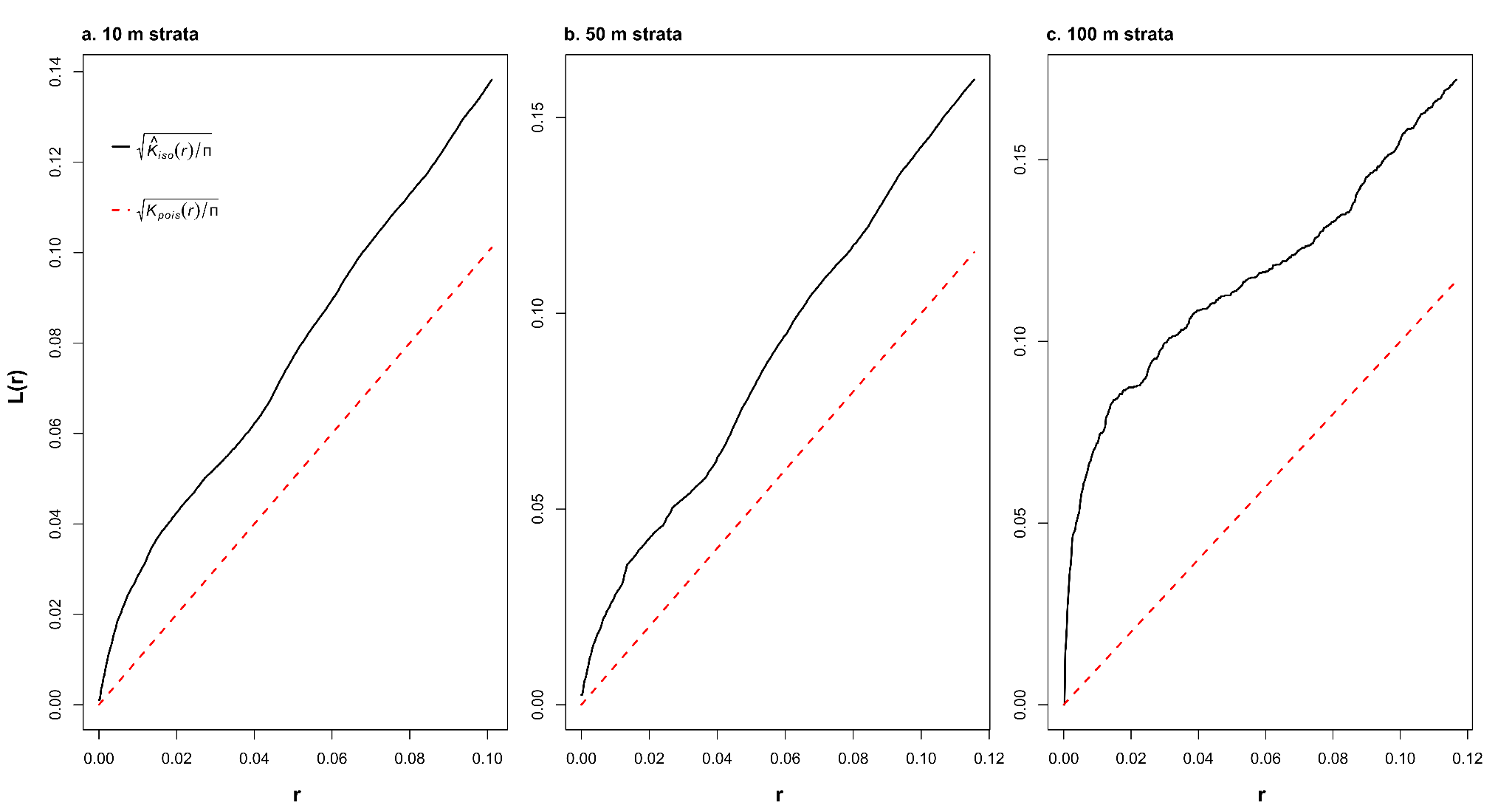


Figure S3. Example of Ripley’s K function calculated for three depth strata for Antarctic krill (*Euphausia superba*) acoustic sampling in the 2021/22 season. The isotropic Ripley’s K (K_iso_, black solid line) is subtracted from the expected poisson process (K_pois_, red dashed line) and averaged for that strata and season. The larger the difference, the more aggregation. Negative values would mean dispersion.


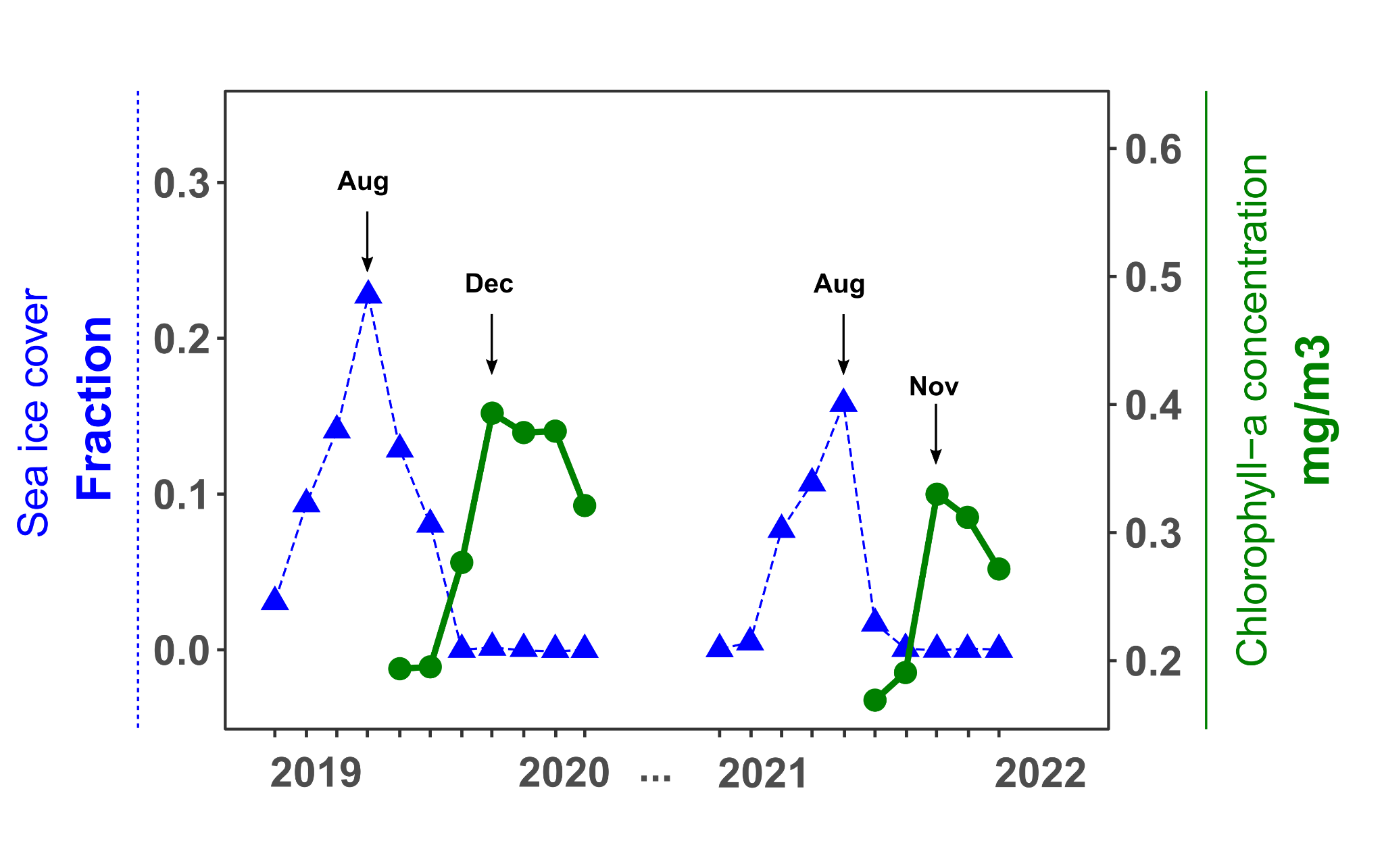


Figure S4. Monthly variability of mean sea ice cover (blue triangles and dashed line) and mean chlorophyll-a concentration (green circles and solid line) in 2019/20 and 2021/22 within a 75 km radius around Harmony Point, Nelson Island (maritime Antarctic Peninsula). Peak of chlorophyll-a in 2021 occurred one month earlier compared to 2019, while the peak of sea ice cover did not vary in time but there was less winter sea ice cover during 2021 compared to 2019.


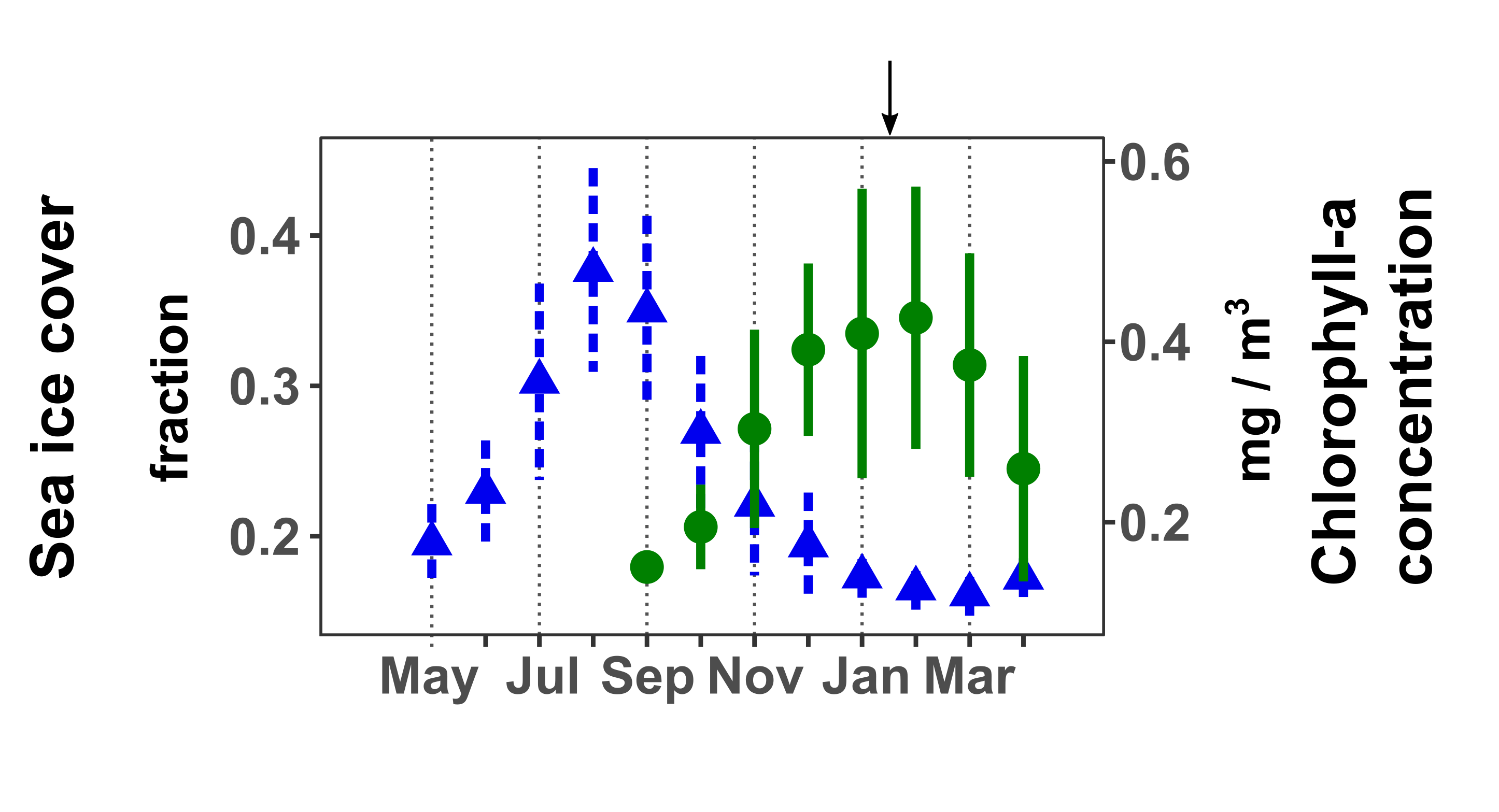


Figure S5. Mean ± sd monthly sea ice cover (blue triangle and dashed lines) and Chlorophyll-a concentration (green circle and solid lines) between May 2005 and April 2022 in the Antarctic Peninsula, subarea 48.1. The arrow above indicates the mean chlorophyll peak in 18 years has been in January and February.
