# Supplementary material for: Contrasting environmental conditions precluded lower availability of Antarctic krill affecting breeding chinstrap penguins in the Antarctic Peninsula
Source: Sci Rep. 2023 Mar 31;13:5265. doi: 10.1038/s41598-023-32352-7 (PMC10066220; doi:10.1038/s41598-023-32352-7)
Supplement: Supplementary file 2 — Supplementary Information 2. [file 41598_2023_32352_MOESM2_ESM.docx]

Supplementary Information

Title: **Contrasting environmental conditions precluded lower availability of Antarctic krill affecting breeding chinstrap penguins in the Antarctic Peninsula**

Nuria Salmerón^1^, Solenne Belle^1^, Francisco Santa Cruz^2^, Nicolás Alegria^3^, Júlia Victória Grohmann Finger^5^, Denyelle Hennayra Corá^5^, Maria Virginia Petry^5^, Cristina Hernández^6^, César A. Cárdenas^2,4^, Lucas Krüger^2,4^*

^1^International Master of Science in Marine Biological Resources (IMBRSea), Ghent University, Krijgslaan 281/S8, Ghent, Belgium.

^2^ Departamento Científico, Instituto Antártico Chileno, Plaza Muñoz Gamero, 1055, Punta Arenas, Chile

^3^ Instituto de Investigación Pesquera (INPESCA), Colón 2780, Talcahuano, Chile

^4^ Millennium Institute Biodiversity of Antarctic and Subantarctic Ecosystems (BASE), Las Palmeras 3425 Ñuñoa, Santiago, Chile

^5^ Laboratório de Ornitologia e Animais Marinhos, Universidade do Vale do Rio dos Sinos (UNISINOS), Av. Unisinos, 950, São Leopoldo, Rio Grande do Sul, Brazil

^6^ Universidad de Magallanes, Avenida Bulnes 01855, Punta Arenas, Chile

*corresponding author: [lkruger@inach.cl](mailto:lkruger@inach.cl) **+56 612 298 100**

R codes for the manuscript

### -------Coding for processing GPS and TDR data-------

library(pracma) # for moving average computation

library(ggplot2)

library(adehabitatLT)

library(lubridate)

library(plyr)

library(patchwork)

### -------TDR and accelerometer processing --------------

library(diveMove)

rm(list=ls(all.names=TRUE))

.rs.restartR()

memory.limit(size=320000000000)

### load TDR data

setwd("D:/DataPinguinos/Nuria/Data/2019-20/ChickRearing/")

df1.1o<-read.csv("HP-6_S1.csv") ###<------------------------------------ CHANGE THIS!!!!!

df1.1<-na.omit(df1.1o[1:8]) # subsetting NA will keep data each second

summary(as.factor(df1.1$Activity))

df1.1$date<-as.POSIXct(strptime(df1.1$Timestamp, format="%d/%m/%Y %H:%M:%S", tz="GMT"))

###GPS data for later

gps1.1<-na.omit(data.frame(Id=c("HPCR06"),###<------------------------------ CHANGE THIS!!!!!!!!

df1.1o[2],df1.1o[3],df1.1o[4],df1.1o[5],

df1.1o[9],df1.1o[10])) ### subsetting only geographical fixes

gps1.1<-subset(gps1.1,location.lat<(-62))

remove(list="df1.1o") #clean memory

gc()#free unused memory

wetcond<-ifelse(df1.1$Activity=="Active/Wet",TRUE,FALSE)

summary(wetcond)

head(df1.1)

### create a TDR object

tdr1.1<-createTDR(time=df1.1$date,depth=df1.1$Depth,dtime=1,speed=F,

concurrentData = df1.1,file="D:/")

dcalib <- calibrateDepth(tdr1.1,

wet.cond = wetcond,

wet.thr=10,

zoc.method="offset",

offset=5,

interp.wet = T,

descent.crit.q=0.01, ascent.crit.q=0,

knot.factor=60)

# examples of dive profiles

plotTDR(dcalib, diveNo=3)

plotTDR(dcalib, diveNo=17)

plotTDR(dcalib, diveNo=27)

plotTDR(dcalib, diveNo=30)

#create a dataframe with dive info

#TDRdf1.1<-data.frame(date=df1.1$date,

# Phases=dcalib@dive.phases,Depth=tdr1.1@depth,

# DiveID=dcalib@dive.activity$dive.id,

#DiveAct=dcalib@dive.activity$dive.activity,

#X=tdr1.1@concurrentData$X,Y=tdr1.1@concurrentData$Y,Z=tdr1.1@concurrentData$Z)

summary(dcalib@dive.activity)

### -------------- summarize dives---------------

TDRm<-diveStats(dcalib,depth.deriv = TRUE)

write.csv(TDRm,"D:/DataPinguinos/Nuria/ProcessedData/TDR/HP06_TDR_CR19.csv") ### <------- CHANGE THIS!!!!--------------

#### -------processing GPS data----------

Home.Lat <- -62.311*pi/180

Home.Long <- -59.212*pi/180

dtime<-as.POSIXct(strptime(gps1.1$Timestamp, format="%d/%m/%Y %H:%M:%S", tz="GMT"))

crs=CRS( "+proj=longlat +datum=WGS84 +no_defs")

coords=coordinates(gps1.1[7:6])

traj1<-as.ltraj(xy=coords,date=dtime,id=gps1.1$Id,

typeII = TRUE,infolocs = gps1.1,

slsp = c("missing"),

proj4string = crs)

plot(traj1)

### resample trip to every second in order to match TDR data

# u = time in seconds

rtraj1<-redisltraj(traj1,u=300,burst=traj1$id,samplex0 = TRUE,type="time",

nnew=100000)

tdf<-ld(rtraj1)

tdf$timeStamp<-as.POSIXct(strptime(tdf$date, format="%Y-%m-%d %H:%M:%S", tz="GMT"))

dat<-tdf

dat$Site<-c("HP")

dat$Lat<-dat$y

dat$Long<-dat$x

Site<-unique(na.omit(unique(dat$Site)))

if(Site=="HP"){

Home.Lat <- -62.311*pi/180

Home.Long <- -59.212*pi/180

}

dat$Distance<-acos(sin(dat$Lat*pi/180)*sin(Home.Lat)+cos(dat$Lat*pi/180)*

cos(Home.Lat)*

cos(Home.Long-dat$Long*pi/180))*(6371*3) #250 m from colony

# now distance between points

n.pos<-length(dat$Lat)

x1<-dat$Lat[-n.pos]

x2<-dat$Lat[-1]

y1<-dat$Long[-n.pos]

y2<-dat$Long[-1]

dat$LegDistance<-c(NA, acos(sin(x1*pi/180)*sin(x2*pi/180)+cos(x1*pi/180)*

cos(x2*pi/180)*

cos(y2*pi/180-y1*pi/180))*(6371*3)) #250m from colony

tdiff<-c(NA, diff(dat$Date)/60) # should be in hours

dat$Speed<-dat$LegDistance/tdiff/3.6 # units are m/s

n.pos<-length(dat$Lat)

dat$Date<-dat$timeStamp

# Now identify trips based on proximity to the Home positions.

# Use a moving average to smooth over arrival/departure and near-landings

MA<-pracma::movavg(dat$Distance, 5, type="s")

last.two<-dat$Distance[c(n.pos-1, n.pos)]

MA<-c(MA[-c(1:2)], last.two) # this centers the 5-pt moving average and

#retains the last two distances for each trip

dat$Home<-ifelse(MA<1, 1, 0)

summary(as.factor(dat$Home))

tail(dat)

# try to identify trips

tt<-cbind(dat$Home, c(dat$Home[1], dat$Home[-n.pos]))

ticker<-1

index<-numeric(n.pos)

for(k in 1:n.pos){

if(k>1){

ticker<-max(index, na.rm=TRUE)

}

index[k]<-ifelse(tt[k,1]==tt[k,2], ticker, ticker+1)

}

index<-ifelse(is.na(index), 0, index)# time consuming command

index<-ifelse(dat$Home==1, -99, index)

dat$Index<-index

index<-na.omit(index[index>0])

n.trips<-length(unique(index))

print(n.trips)

if(n.trips>0){

ttt<-data.frame(Index=unique(index), Trip=1:n.trips)

dat<-merge(dat, ttt, by="Index", all=TRUE)

} else {

dat$Trip <-rep(1, n.pos)

}

# reorder the data

dat<-dat[order(dat$Date),]

summary(as.factor(dat$Trip))

dat.h<-subset(dat,Trip=="1"|Trip=="2"|Trip=="3"|Trip=="4"|Trip=="5"|Trip=="6"|Trip=="7"|

Trip=="9"|Trip=="10"|Trip=="11")

###<--------------------- CHANGE THIS!!!!!!!!!!!!!!!!

datH<-na.omit(data.frame(date=dat.h$timeStamp,Dist=dat.h$Distance,

LegDist=dat.h$LegDistance,Trip=dat.h$Trip,Lat=dat.h$Lat,Long=dat.h$Long))

summary(datH$LegDist)

datH<-subset(datH,LegDist<3)

ggplot(datH,aes(date,LegDist))+geom_point()

write.csv(datH,"D:/DataPinguinos/Nuria/ProcessedData/GPS/HP03_GPS_CR19.csv") ###<----- CHANGE THIS!!!!!!!!!!!!!!!!

summary(as.factor(datH$Trip))

###------trips summary all together----------

trips.summary<-ddply(datH, c("Trip"), summarise,

start = min(date),

end=max(date),

cumDist = sum(LegDist) )

trips.summary$start[1]

### calculate decitimes

trips.summary$startDec<-decimal_date(trips.summary$start)

trips.summary$endDec<-decimal_date(trips.summary$end)

trips.summary$duration<-(trips.summary$end-trips.summary$start)

### change numbers according to the number of trips

trips.summary$Trip[trips.summary$Trip=="1"]<-1

trips.summary$Trip[trips.summary$Trip=="2"]<-2

trips.summary$Trip[trips.summary$Trip=="3"]<-3

trips.summary$Trip[trips.summary$Trip=="4"]<-4

trips.summary$Trip[trips.summary$Trip=="5"]<-5

trips.summary$Trip[trips.summary$Trip=="6"]<-6

trips.summary$Trip[trips.summary$Trip=="7"]<-7

trips.summary$Trip[trips.summary$Trip=="9"]<-8

trips.summary$Trip[trips.summary$Trip=="10"]<-9

trips.summary$Trip[trips.summary$Trip=="11"]<-10

trips.summary$Trip[trips.summary$Trip=="13"]<-11

trips.summary$Trip[trips.summary$Trip=="14"]<-12

trips.summary$Trip[trips.summary$Trip=="15"]<-13

trips.summary

### TDR

head(TDRm)

TDRm$start<-as.POSIXct(strptime(TDRm$begdesc, format="%Y-%m-%d %H:%M:%S", tz="GMT")) #begin descent

TDRm$startDec<-decimal_date(TDRm$start)

TDRm$end<-as.POSIXct(strptime(TDRm$begasc, format="%Y-%m-%d %H:%M:%S", tz="GMT")) #begin ascent

TDRm$endDec<-decimal_date(TDRm$end)

head(TDRm)

### change numbers according to the number of trips

TDRm$Trip[TDRm$startDec>trips.summary$startDec[1] & TDRm$endDec<trips.summary$endDec[1]]<-1

TDRm$Trip[TDRm$startDec>trips.summary$startDec[2] & TDRm$endDec<trips.summary$endDec[2]]<-2

TDRm$Trip[TDRm$startDec>trips.summary$startDec[3] & TDRm$endDec<trips.summary$endDec[3]]<-3

TDRm$Trip[TDRm$startDec>trips.summary$startDec[4] & TDRm$endDec<trips.summary$endDec[4]]<-4

TDRm$Trip[TDRm$startDec>trips.summary$startDec[5] & TDRm$endDec<trips.summary$endDec[5]]<-5

TDRm$Trip[TDRm$startDec>trips.summary$startDec[6] & TDRm$endDec<trips.summary$endDec[6]]<-6

TDRm$Trip[TDRm$startDec>trips.summary$startDec[7] & TDRm$endDec<trips.summary$endDec[7]]<-7

TDRm$Trip[TDRm$startDec>trips.summary$startDec[8] & TDRm$endDec<trips.summary$endDec[8]]<-8

TDRm$Trip[TDRm$startDec>trips.summary$startDec[9] & TDRm$endDec<trips.summary$endDec[9]]<-9

TDRm$Trip[TDRm$startDec>trips.summary$startDec[10] & TDRm$endDec<trips.summary$endDec[10]]<-10

TDRm$Trip[TDRm$startDec>trips.summary$startDec[11] & TDRm$endDec<trips.summary$endDec[11]]<-11

TDRm$Trip[TDRm$startDec>trips.summary$startDec[12] & TDRm$endDec<trips.summary$endDec[12]]<-12

TDRm$Trip[TDRm$startDec>trips.summary$startDec[13] & TDRm$endDec<trips.summary$endDec[13]]<-13

summary(as.factor(TDRm$Trip))

head(TDRm)

TDRm2<-(subset(TDRm,Trip=="1"|Trip=="2"|Trip=="3"|Trip=="4"|Trip=="5"|

Trip=="6"|Trip=="7"|Trip=="8"|Trip=="9"|Trip=="10"))

head(TDRm2)

summary(as.factor(TDRm2$Trip))

dives<-ddply(TDRm2, c("Trip"), summarise,

NCDiv=length(na.omit(botttim)), #number of complete dives

NDiv=length(Trip), #number of dives without bottom phase

SBotT=sum(na.omit(botttim)), # accumulated bottom time

SDiveDur=sum(divetim), # total dive duration

MdiveDur=mean(divetim),#mean dive duration

BDepM=mean(na.omit(bottdep.mean)), #mean bottom phase depth

BDepMax=max(na.omit(maxdep)), #maximum bottom phase depth

WigS=sum(na.omit(bottdist)), ### total amount of wiggling (capture effort)

WigM=mean(na.omit(bottdist))) ### number of wiggling per dive

dives

trips.summary

trips.dives<-merge(trips.summary,dives)

trips.dives

write.csv(trips.dives,"D:/DataPinguinos/Nuria/ProcessedData/trips.dives_HP_03_CR19.csv") ### <---------------CHANGE THIS!!!!--------------

rm(list=ls(all.names=TRUE))

.rs.restartR()

### -------Coding for joining GPS and TDR data-------

library(pracma) # for moving average computation

library(ggplot2)

library(adehabitatLT)

library(lubridate)

library(plyr)

###load GPS data

setwd("D:/DataPinguinos/Nuria/ProcessedData/GPS/")

INC19.03<-data.frame(read.csv("HP03_GPS_INC19.csv"), ID="HP03", Period="IB19")

INC19.06<-data.frame(read.csv("HP06_GPS_INC19.csv"), ID="HP06", Period="IB19")

INC19.07<-data.frame(read.csv("HP07_GPS_INC19.csv"), ID="HP07", Period="IB19")

INC19.09<-data.frame(read.csv("HP09_GPS_INC19.csv"), ID="HP09", Period="IB19")

INC19.11<-data.frame(read.csv("HP11_GPS_INC19.csv"), ID="HP11", Period="IB19")

INC19.16<-data.frame(read.csv("HP16_GPS_INC19.csv"), ID="HP16", Period="IB19")

INC19.17<-data.frame(read.csv("HP17_GPS_INC19.csv"), ID="HP17", Period="IB19")

INC19.18<-data.frame(read.csv("HP18_GPS_INC19.csv"), ID="HP18", Period="IB19")

CR19.03<-data.frame(read.csv("HP03_GPS_CR19.csv"), ID="HP03", Period="CR19")

CR19.06<-data.frame(read.csv("HP06_GPS_CR19.csv"), ID="HP06", Period="CR19")

CR19.07<-data.frame(read.csv("HP07_GPS_CR19.csv"), ID="HP07", Period="CR19")

CR19.10<-data.frame(read.csv("HP10_GPS_CR19.csv"), ID="HP10", Period="CR19")

CR19.11<-data.frame(read.csv("HP11_GPS_CR19.csv"), ID="HP11", Period="CR19")

CR19.14<-data.frame(read.csv("HP14_GPS_CR19.csv"), ID="HP14", Period="CR19")

CR19.17<-data.frame(read.csv("HP17_GPS_CR19.csv"), ID="HP17", Period="CR19")

CR19.19<-data.frame(read.csv("HP19_GPS_CR19.csv"), ID="HP19", Period="CR19")

CR19.20<-data.frame(read.csv("HP20_GPS_CR19.csv"), ID="HP20", Period="CR19")

CR21.03<-data.frame(read.csv("HP03_GPS_CR22.csv"), ID="HP03", Period="IB21")

CR21.06<-data.frame(read.csv("HP06_GPS_CR22.csv"), ID="HP06", Period="IB21")

CR21.07<-data.frame(read.csv("HP07_GPS_CR22.csv"), ID="HP07", Period="IB21")

CR21.08<-data.frame(read.csv("HP08_GPS_CR22.csv"), ID="HP08", Period="IB21")

CR21.11<-data.frame(read.csv("HP11_GPS_CR22.csv"), Period="IB21")

CR21.16<-data.frame(read.csv("HP16_GPS_CR22.csv"), ID="HP16", Period="IB21")

CR21.19<-data.frame(read.csv("HP19_GPS_CR22.csv"), ID="HP19", Period="IB21")

CR21.20<-data.frame(read.csv("HP20_GPS_CR22.csv"), ID="HP20", Period="IB21")

tracks<-rbind(INC19.03,INC19.06,INC19.07,INC19.09,INC19.11,INC19.16,INC19.17,INC19.18,

CR19.03,CR19.06,CR19.07,CR19.10,CR19.11,CR19.14[2:10],CR19.17[2:10],CR19.19,CR19.20[2:10],

CR21.03,CR21.06,CR21.07,CR21.08,CR21.11[2:10],CR21.16,CR21.19,CR21.20)

head(tracks)

write.csv(tracks,"AllTracks.csv")

#### load TDR dive calibrated data

setwd("D:/DataPinguinos/Nuria/ProcessedData/TDR/")

INC19.03.td<-data.frame(read.csv("HP03_TDR_INC19.csv"), ID="HP03", Period="INC19")

INC19.06.td<-data.frame(read.csv("HP06_TDR_INC19.csv"), ID="HP06", Period="INC19")

INC19.07.td<-data.frame(read.csv("HP07_TDR_INC19.csv"), ID="HP07", Period="INC19")

INC19.09.td<-data.frame(read.csv("HP09_TDR_INC19.csv"), ID="HP09", Period="INC19")

INC19.11.td<-data.frame(read.csv("HP11_TDR_INC19.csv"), ID="HP11", Period="INC19")

INC19.16.td<-data.frame(read.csv("HP16_TDR_INC19.csv"), ID="HP16", Period="INC19")

INC19.17.td<-data.frame(read.csv("HP17_TDR_INC19.csv"), ID="HP17", Period="INC19")

INC19.18.td<-data.frame(read.csv("HP18_TDR_INC19.csv"), ID="HP18", Period="INC19")

CR19.03.td<-data.frame(read.csv("HP03_TDR_CR19.csv"), ID="HP03",Period="CR19")

CR19.06.td<-data.frame(read.csv("HP06_TDR_CR19.csv"), ID="HP06",Period="CR19")

CR19.07.td<-data.frame(read.csv("HP07_TDR_CR19.csv"), ID="HP07",Period="CR19")

CR19.10.td<-data.frame(read.csv("HP10_TDR_CR19.csv"), ID="HP10",Period="CR19")

CR19.11.td<-data.frame(read.csv("HP11_TDR_CR19.csv"), ID="HP11",Period="CR19")

CR19.14.td<-data.frame(read.csv("HP14_TDR_CR19.csv"), ID="HP14",Period="CR19")

CR19.17.td<-data.frame(read.csv("HP17_TDR_CR19.csv"), ID="HP17",Period="CR19")

CR19.19.td<-data.frame(read.csv("HP19_TDR_CR19.csv"), ID="HP19",Period="CR19")

CR19.20.td<-data.frame(read.csv("HP20_TDR_CR19.csv"), ID="HP20",Period="CR19")

CR21.03.td<-data.frame(read.csv("HP03_TDR_CR21.csv"), ID="HP03",Period="CR21")

CR21.06.td<-data.frame(read.csv("HP06_TDR_CR21.csv"), ID="HP06",Period="CR21")

CR21.07.td<-data.frame(read.csv("HP07_TDR_CR21.csv"), ID="HP07",Period="CR21")

CR21.08.td<-data.frame(read.csv("HP08_TDR_CR21.csv"), ID="HP08",Period="CR21")

CR21.11.td<-data.frame(read.csv("HP11_TDR_CR21.csv"), ID="HP11",Period="CR21")

CR21.16.td<-data.frame(read.csv("HP16_TDR_CR21.csv"), ID="HP16",Period="CR21")

CR21.19.td<-data.frame(read.csv("HP19_TDR_CR21.csv"), ID="HP19",Period="CR21")

CR21.20.td<-data.frame(read.csv("HP20_TDR_CR21.csv"), ID="HP20",Period="CR21")

### summarize GPS data

summary(as.factor(INC19.18$Trip)) ####### -----------------------CHANGE-------

Trip<-INC19.18 ####### ------------------------------------------CHANGE---------

Trip$date<-as.POSIXct(strptime(Trip$date, format="%Y-%m-%d %H:%M:%S", tz="GMT"))

trips.summary<-ddply(Trip, c("Trip"), summarise,

start = as.POSIXct(strptime(min(date), format="%Y-%m-%d %H:%M:%S", tz="GMT")),

end=as.POSIXct(strptime(max(date), format="%Y-%m-%d %H:%M:%S", tz="GMT")),

cumDist = sum(LegDist))

trips.summary$startDec<-decimal_date(trips.summary$start)

trips.summary$endDec<-decimal_date(trips.summary$end)

trips.summary$duration<-(trips.summary$end-trips.summary$start)

trips.summary

### only for correcting non-sequential number of trips , not all data have it

trips.summary$Trip[trips.summary$Trip=="3"]<-1

trips.summary$Trip[trips.summary$Trip=="6"]<-2

trips.summary$Trip[trips.summary$Trip=="7"]<-3

trips.summary$Trip[trips.summary$Trip=="8"]<-4

trips.summary$Trip[trips.summary$Trip=="10"]<-5

trips.summary$Trip[trips.summary$Trip=="11"]<-6

trips.summary$Trip[trips.summary$Trip=="12"]<-7

trips.summary$Trip[trips.summary$Trip=="13"]<-8

trips.summary$Trip[trips.summary$Trip=="14"]<-9

trips.summary$Trip[trips.summary$Trip=="15"]<-10

trips.summary$Trip[trips.summary$Trip=="16"]<-11

trips.summary

### summarize TDR data

TDRm<-INC19.18.td ####### ------------------------------------------------CHANGE---------

head(TDRm)

TDRm$start<-as.POSIXct(strptime(TDRm$begdesc, format="%Y-%m-%d %H:%M:%S", tz="GMT")) #begin descent

TDRm$startDec<-decimal_date(TDRm$start)

TDRm$end<-as.POSIXct(strptime(TDRm$begasc, format="%Y-%m-%d %H:%M:%S", tz="GMT")) #begin ascent

TDRm$endDec<-decimal_date(TDRm$end)

head(TDRm)

### identify number of trips on TDR data

TDRm$Trip[TDRm$startDec>trips.summary$startDec[1] & TDRm$endDec<trips.summary$endDec[1]]<-1

TDRm$Trip[TDRm$startDec>trips.summary$startDec[2] & TDRm$endDec<trips.summary$endDec[2]]<-2

TDRm$Trip[TDRm$startDec>trips.summary$startDec[3] & TDRm$endDec<trips.summary$endDec[3]]<-3

TDRm$Trip[TDRm$startDec>trips.summary$startDec[4] & TDRm$endDec<trips.summary$endDec[4]]<-4

TDRm$Trip[TDRm$startDec>trips.summary$startDec[5] & TDRm$endDec<trips.summary$endDec[5]]<-5

TDRm$Trip[TDRm$startDec>trips.summary$startDec[6] & TDRm$endDec<trips.summary$endDec[6]]<-6

TDRm$Trip[TDRm$startDec>trips.summary$startDec[7] & TDRm$endDec<trips.summary$endDec[7]]<-7

TDRm$Trip[TDRm$startDec>trips.summary$startDec[8] & TDRm$endDec<trips.summary$endDec[8]]<-8

TDRm$Trip[TDRm$startDec>trips.summary$startDec[9] & TDRm$endDec<trips.summary$endDec[9]]<-9

TDRm$Trip[TDRm$startDec>trips.summary$startDec[10] & TDRm$endDec<trips.summary$endDec[10]]<-10

TDRm$Trip[TDRm$startDec>trips.summary$startDec[11] & TDRm$endDec<trips.summary$endDec[11]]<-11

TDRm$Trip[TDRm$startDec>trips.summary$startDec[12] & TDRm$endDec<trips.summary$endDec[12]]<-12

TDRm$Trip[TDRm$startDec>trips.summary$startDec[13] & TDRm$endDec<trips.summary$endDec[13]]<-13

summary(as.factor(TDRm$Trip))

TDRm2<-(subset(TDRm,Trip=="1"|Trip=="2"|Trip=="3"|Trip=="4"|Trip=="5"|

Trip=="6"|Trip=="7"|Trip=="8"|Trip=="9"|Trip=="10"|

Trip=="11"|Trip=="12"|Trip=="13"))

summary(as.factor(TDRm2$Trip))

ggplot(TDRm,aes(startDec,bottdist))+geom_point()

#summary of dives for each trip

dives<-ddply(TDRm2, c("Trip"), summarise,

NCDiv=length(na.omit(botttim)), #number of complete dives

NDiv=length(Trip), #number of dives without bottom phase

SBotT=sum(na.omit(botttim)), # accumulated bottom time

SDiveDur=sum(divetim), # total dive duration

MdiveDur=mean(divetim),#mean dive duration

BDepM=mean(na.omit(bottdep.mean)), #mean bottom phase depth

BDepMax=max(na.omit(maxdep)), #maximum bottom phase depth

WigS=sum(na.omit(bottdist)), ### total amount of wiggling (capture effort)

WigM=mean(na.omit(bottdist))) ### number of wiggling per dive

dives

trips.summary

trips.dives<-merge(trips.summary,dives)

trips.dives

write.csv(trips.dives,"D:/DataPinguinos/Nuria/ProcessedData/INC19.18.td.csv") ####### -----------CHANGE--------

### ------------------Kernel Usage Density--------------------

library(adehabitatHR)

library(raster)

library(udunits2)

head(tracks)

setwd("D:/DataPinguinos/Nuria/ProcessedData/GPS/")

xy<-coordinates(data.frame(tracks$Long,tracks$Lat))

ID<-data.frame(ID=paste(tracks$ID,tracks$Period,sep="-"))

head(ID)

spdf<-SpatialPointsDataFrame(xy,ID)

KUD<-kernelUD(spdf, h = 0.01, grid = 800,same4all = TRUE, kern = c("epa"), extent=0.1)

image(KUD)

vud <- getvolumeUD(KUD)

image(vud)

vud <- getvolumeUD(KUD)

vud1<-raster(as.image.SpatialGridDataFrame(vud[[1]]))

vud2<-raster(as.image.SpatialGridDataFrame(vud[[2]]))

vud3<-raster(as.image.SpatialGridDataFrame(vud[[3]]))

vud4<-raster(as.image.SpatialGridDataFrame(vud[[4]]))

vud5<-raster(as.image.SpatialGridDataFrame(vud[[5]]))

vud6<-raster(as.image.SpatialGridDataFrame(vud[[6]]))

vud7<-raster(as.image.SpatialGridDataFrame(vud[[7]]))

vud8<-raster(as.image.SpatialGridDataFrame(vud[[8]]))

vud9<-raster(as.image.SpatialGridDataFrame(vud[[9]]))

vud10<-raster(as.image.SpatialGridDataFrame(vud[[10]]))

vud11<-raster(as.image.SpatialGridDataFrame(vud[[11]]))

vud12<-raster(as.image.SpatialGridDataFrame(vud[[12]]))

vud13<-raster(as.image.SpatialGridDataFrame(vud[[13]]))

vud14<-raster(as.image.SpatialGridDataFrame(vud[[14]]))

vud15<-raster(as.image.SpatialGridDataFrame(vud[[15]]))

vud16<-raster(as.image.SpatialGridDataFrame(vud[[16]]))

vud17<-raster(as.image.SpatialGridDataFrame(vud[[17]]))

vud18<-raster(as.image.SpatialGridDataFrame(vud[[18]]))

vud19<-raster(as.image.SpatialGridDataFrame(vud[[19]]))

vud20<-raster(as.image.SpatialGridDataFrame(vud[[20]]))

vud21<-raster(as.image.SpatialGridDataFrame(vud[[21]]))

vud22<-raster(as.image.SpatialGridDataFrame(vud[[22]]))

vud23<-raster(as.image.SpatialGridDataFrame(vud[[23]]))

vud24<-raster(as.image.SpatialGridDataFrame(vud[[24]]))

vudinc<-stack(vud1,vud2,vud3,vud4,vud5,vud6,vud7,vud8,vud9,vud10,

vud11,vud12,vud13,vud14,vud15,vud16,vud17,vud18,vud19,vud20,

vud21,vud22,vud23,vud24,vud25)

volud<-calc(vudinc,fun=mean)

min(volud)

image(volud)

plot(volud)

raster::writeRaster(volud,"All_VUD.tif",overwrite=T)

### ---------- compare 2019/20 and 2021/22----------------

rm(list=ls(all.names=TRUE))

.rs.restartR()

library(pracma) # for moving average computation

library(ggplot2)

library(adehabitatLT)

library(lubridate)

library(plyr)

library(patchwork)

library(ggplot2)

library(ggExtra)

library(gridExtra)

library(grid)

th<-theme(axis.text=element_text(size=14, face="bold",colour="grey30"),

axis.title=element_text(size=16,face="bold"),

legend.text = element_text(size=16),

plot.title=element_text(size=14),

panel.grid.major = element_blank(),

panel.grid.minor = element_blank(),

panel.background = element_blank())

#### ---------- Breeding success 2019/20 and 2021/22----------------

sc<-read.csv("D:/DataPinguinos/Nuria/Data/Successeca5658.csv")

library(sjPlot)

ggplot(sc,aes(Year, Success))+geom_boxplot()+

theme_bw()+th+ylim(0.4,1.6)+ylab("Chicks raised per nest")

shapiro.test(sc$Success)

bartlett.test(sc$Success,sc$Year)

lmn<-lm(Success~Year,data=sc)

summary(lmn)

plot_model(lmn,type="pred")

### -------Load Trip-Dive data --------------

setwd("D:/DataPinguinos/Nuria/ProcessedData/")

### season 2019/20

### incubation

INC19.03<-data.frame(read.csv("INC19.03.td.csv"), ID="INC19/20_HP03")

INC19.06<-data.frame(read.csv("INC19.06.td.csv"), ID="INC19/20_HP06")

INC19.07<-data.frame(read.csv("INC19.07.td.csv"), ID="INC19/20_HP07")

INC19.09<-data.frame(read.csv("INC19.09.td.csv"), ID="INC19/20_HP09")

INC19.11<-data.frame(read.csv("INC19.11.td.csv"), ID="INC19/20_HP11")

INC19.16<-data.frame(read.csv("INC19.16.td.csv"), ID="INC19/20_HP16")

INC19.17<-data.frame(read.csv("INC19.17.td.csv"), ID="INC19/20_HP17")

INC19.18<-data.frame(read.csv("INC19.18.td.csv"), ID="INC19/20_HP18")

CR19.03<-data.frame(read.csv("CR19.03.td.csv"), ID="CR19/20_HP03")

CR19.06<-data.frame(read.csv("CR19.06.td.csv"), ID="CR19/20_HP06")

CR19.07<-data.frame(read.csv("CR19.07.td.csv"), ID="CR19/20_HP07")

CR19.10<-data.frame(read.csv("CR19.10.td.csv"), ID="CR19/20_HP10")

CR19.11<-data.frame(read.csv("CR19.11.td.csv"), ID="CR19/20_HP11")

CR19.14<-data.frame(read.csv("CR19.14.td.csv"), ID="CR19/20_HP14")

CR19.17<-data.frame(read.csv("CR19.17.td.csv"), ID="CR19/20_HP17")

CR19.19<-data.frame(read.csv("CR19.19.td.csv"), ID="CR19/20_HP19")

CR19.20<-data.frame(read.csv("CR19.20.td.csv"), ID="CR19/20_HP20")

CR21.03<-data.frame(read.csv("CR21.03.td.csv"), ID="CR21/20_HP03")

CR21.06<-data.frame(read.csv("CR21.06.td.csv"), ID="CR21/20_HP06")

CR21.07<-data.frame(read.csv("CR21.07.td.csv"), ID="CR21/20_HP07")

CR21.08<-data.frame(read.csv("CR21.08.td.csv"), ID="CR21/20_HP08")

CR21.11<-data.frame(read.csv("CR21.11.td.csv"), ID="CR21/20_HP11")

CR21.16<-data.frame(read.csv("CR21.16.td.csv"), ID="CR21/20_HP16")

CR21.19<-data.frame(read.csv("CR21.19.td.csv"), ID="CR21/20_HP19")

CR21.20<-data.frame(read.csv("CR21.20.td.csv"), ID="CR21/20_HP20")

inc19.20<-rbind(INC19.03,INC19.06,INC19.07,INC19.09,INC19.11,INC19.16,INC19.17,INC19.18)

cr19.20<-rbind(CR19.03,CR19.06,CR19.07,CR19.10,CR19.11,CR19.14,CR19.17,CR19.19,CR19.20)

cr21.22<-rbind( CR21.03,CR21.06,CR21.07,CR21.08,CR21.11,CR21.16,CR21.19,CR21.20)

#exclude outliers

inc19.20<-subset(inc19.20,duration<30)

cr19.20<-subset(cr19.20,duration<30)

cr21.22<-subset(cr21.22,duration<30)

inc19.20$start<-as.POSIXct(strptime(inc19.20$start, format="%Y-%m-%d %H:%M:%S", tz="GMT"))

inc19.20$end<-as.POSIXct(strptime(inc19.20$end, format="%Y-%m-%d %H:%M:%S", tz="GMT"))

cr19.20$start<-as.POSIXct(strptime(cr19.20$start, format="%Y-%m-%d %H:%M:%S", tz="GMT"))

cr19.20$end<-as.POSIXct(strptime(cr19.20$end, format="%Y-%m-%d %H:%M:%S", tz="GMT"))

cr21.22$start<-as.POSIXct(strptime(cr21.22$start, format="%Y-%m-%d %H:%M:%S", tz="GMT"))

cr21.22$end<-as.POSIXct(strptime(cr21.22$end, format="%Y-%m-%d %H:%M:%S", tz="GMT"))

chdf<-rbind(inc19.20,cr19.20,cr21.22)

chdf$start<-as.POSIXct(strptime(chdf$start, format="%Y-%m-%d %H:%M:%S", tz="GMT"))

chdf$end<-as.POSIXct(strptime(chdf$end, format="%Y-%m-%d %H:%M:%S", tz="GMT"))

head(chdf)

chdf$Season<-substring(chdf$ID,first=1,last=8)

chdf$Season<-factor(chdf$Season,levels=c("INC19/20","CR19/20_","CR21/20_"))

chdf$Season2=ifelse(chdf$startDec<2021,"2019/20","2021/22")

### summary

ddply(chdf, c("Season2"), summarise,

CDis=mean(cumDist),

CDissd=sd(cumDist),

DUR=mean(duration),

DURsd=sd(duration),

CD=mean(NCDiv/NDiv),CDsd=sd(NCDiv/NDiv),

MDD=mean(MdiveDur),MDDsd=sd(MdiveDur),

SDD=mean(SDiveDur),SDDsd=sd(SDiveDur),

BDM=mean(BDepM),BDMsd=sd(BDepM),

BMAX=mean(BDepMax),BMAXsd=sd(BDepMax),

WIG=mean(WigM),WIGsd=sd(WigM))

ggplot(chdf,aes(Season2,duration))+geom_boxplot()+

ylab("Hours")+theme_bw()+th+xlab("")+

labs(title="a. Foraging trip duration",subtitle="F1,176=20.36, P<0.001")+

ggplot(chdf,aes(Season2,cumDist))+geom_boxplot()+

ylab("Kilometers")+theme_bw()+th+xlab("")+

labs(title="b. Foraging trip cumulative distance",subtitle="F1,176=4.58, P=0.033")+

ggplot(chdf,aes(Season2,NCDiv/NDiv))+geom_boxplot()+

ylab("Proportion")+theme_bw()+th+xlab("")+

labs(title="c. Frequency of complete dives",subtitle="F1,176=17.37, P<0.001")+

ggplot(chdf,aes(Season2,MdiveDur/60))+geom_boxplot()+

ylab("Minutes")+theme_bw()+th+xlab("")+

labs(title="d. Mean dive duration",subtitle="F1,176=0.47, P=0.514")+

ggplot(chdf,aes(Season2,SDiveDur/60))+geom_boxplot()+

ylab("Minutes")+theme_bw()+th+xlab("")+

labs(title="e. Cumulative dive duration",subtitle="F1,176=28.82, P<0.001")+

ggplot(chdf,aes(Season2,BDepM))+geom_boxplot()+

ylab("Meters")+theme_bw()+th+xlab("")+

labs(title="f. Bottom phase mean depth",subtitle="F1,176=0.55, P=0.463")+

ggplot(chdf,aes(Season2,BDepMax))+geom_boxplot()+

ylab("Meters")+theme_bw()+th+xlab("")+

labs(title="g. Bottom phase max depth",subtitle="F1,176=14.532, P<0.001")+

ggplot(chdf,aes(Season2,WigM))+geom_boxplot()+

ylab("Wiggles")+theme_bw()+th+xlab("Season")+

labs(title="h. Mean capture effort",subtitle="F1,176=18.36, P<0.001")+

ggplot(sc,aes(Year, Success))+geom_boxplot()+xlab("Season")+

theme_bw()+th+ylim(0.4,1.6)+ylab("Chicks raised per nest")+

labs(title="i. Breeding success",subtitle="F1,22=22.92, P<0.001")

### compare data

head(chdf)

library(PERMANOVA)

###trip duration

Xtd=IniTransform(as.matrix(chdf$duration),

transform="Standardize columns")

TD=DistContinuous(Xtd)

ftd<-PERMANOVA(TD,as.factor(mfdf$Season),nperm=999)

print(ftd)$Initial$Global

### cummulative distance

Xcd=IniTransform(as.matrix(chdf$cumDist),

transform="Standardize columns")

TC=DistContinuous(Xcd)

ftc<-PERMANOVA(TC,as.factor(mfdf$Season),nperm=999)

print(ftc)

### prop complete dives

head(chdf)

Xnd=IniTransform(as.matrix(chdf$NCDiv/chdf$NDiv),

transform="Standardize columns")

Tnd=DistContinuous(Xnd)

ftcnd<-PERMANOVA(Tnd,as.factor(mfdf$Season),nperm=999)

print(ftcnd)

### mean dive duration

head(chdf)

Xmd=IniTransform(as.matrix(chdf$MdiveDur),

transform="Standardize columns")

Tmd=DistContinuous(Xmd)

ftmd<-PERMANOVA(Tmd,as.factor(mfdf$Season),nperm=999)

print(ftmd)

### cumulative dive duration

head(chdf)

Xsd=IniTransform(as.matrix(chdf$SDiveDur),

transform="Standardize columns")

Tsd=DistContinuous(Xsd)

ftsd<-PERMANOVA(Tsd,as.factor(mfdf$Season),nperm=999)

print(ftsd)

### bottom phase mean depth

head(chdf)

Xbd=IniTransform(as.matrix(chdf$BDepM),

transform="Standardize columns")

Tbd=DistContinuous(Xbd)

ftbd<-PERMANOVA(Tbd,as.factor(mfdf$Season),nperm=999)

print(ftbd)

### bottom phase max depth

head(chdf)

Xmd=IniTransform(as.matrix(chdf$BDepMax),

transform="Standardize columns")

Tmd=DistContinuous(Xmd)

ftmd<-PERMANOVA(Tmd,as.factor(mfdf$Season),nperm=999)

print(ftmd)

### mean capture effort

head(chdf)

Xme=IniTransform(as.matrix(chdf$WigM),

transform="Standardize columns")

Tme=DistContinuous(Xme)

ftme<-PERMANOVA(Tme,as.factor(mfdf$Season),nperm=999)

print(ftme)

### total capture effort

head(chdf)

Xte=IniTransform(as.matrix(chdf$WigS),

transform="Standardize columns")

Tte=DistContinuous(Xte)

ftte<-PERMANOVA(Tte,as.factor(mfdf$Season),nperm=999)

summary(ftte)

-------------Environmental Analysis--------------------------

library(ggplot2)

library(lubridate)

library(tidyr)

library(tidyquant)

library(dplyr)

library(broom)

library(purrr)

library(stringr)

library(knitr)

library(timetk)

library(reshape2)

library(plyr)

library(patchwork)

library(dynlm)

library(raster)

library(reshape2)

th<- theme(axis.text=element_text(size=12, face="bold",colour="grey30"),

axis.title=element_text(size=12,face="bold"),

legend.text = element_text(size=12),

panel.grid.major = element_blank(),

panel.grid.minor = element_blank(),

title =element_text(size=12, face="bold",colour="black"),

panel.spacing = unit(1, "lines")) # theme for plots

### load environmental data

# data were downloaded from Giovanni NASA browser https://giovanni.gsfc.nasa.gov/giovanni

# using

# variables were:

# CHL Chlorophyll-a concentration (mg/m3)

#NASA Goddard Space Flight Center, Ocean Ecology Laboratory, Ocean Biology Processing Group. #

#Moderate-resolution Imaging Spectroradiometer (MODIS) Aqua Chlorophyll Data; 2018 Reprocessing.

#NASA OB.DAAC, Greenbelt, MD, USA. doi: 10.5067/AQUA/MODIS/L3M/CHL/2018.

#Accessed on July/29/2022

# PAR Photossintetically available radiation ()

#NASA Goddard Space Flight Center, Ocean Ecology Laboratory, Ocean Biology Processing Group.

#Moderate-resolution Imaging Spectroradiometer (MODIS) Aqua Photosynthetically Available Radiation Data;

#2018 Reprocessing. NASA OB.DAAC, Greenbelt, MD, USA. doi: 10.5067/AQUA/MODIS/L3M/PAR/2018.

#Accessed on July/29/2022

#SIC Sea Ice Cover (fraction of tile)

#Global Modeling and Assimilation Office (GMAO) (2015),

#MERRA-2 tavgM_2d_flx_Nx: 2d,Monthly mean,Time-Averaged,Single-Level,Assimilation,Surface Flux Diagnostics V5.12.4,

#Greenbelt, MD, USA, Goddard Earth Sciences Data and Information Services Center (GES DISC),

#Accessed: [July/29/2022], 10.5067/0JRLVL8YV2Y4

# surface wind speed (m/s)

#Global Modeling and Assimilation Office (GMAO) (2015),

#MERRA-2 tavgM_2d_flx_Nx: 2d,Monthly mean,Time-Averaged,Single-Level,Assimilation,Surface Flux Diagnostics V5.12.4,

#Greenbelt, MD, USA, Goddard Earth Sciences Data and Information Services Center (GES DISC),

#Accessed: [July/29/2022], 10.5067/0JRLVL8YV2Y4

envi<-read.csv("D:/DataPinguinos/Nuria/Data/ENVI_TimeSeries/envi.csv")

head(envi)

### create time series xts

envi$timestamp<-as.POSIXct(strptime(envi$time, format="%d-%m-%Y", tz="GMT"))

#month and year from the time stamp

envi$Month<-month(envi$timestamp)

envi$year<-year(envi$timestamp)

# calculate mean and sd for each month between 2005 and 2022

enviM<-ddply(envi, c("Month"), summarise,

CHL=mean(na.omit(CHLA)),

CHLsd=sd(na.omit(CHLA)),

SIC=mean(FRSIC),

SICsd=sd(FRSIC),

WS=mean(Wspeed),

WSsd=sd(Wspeed),

PAR=mean(na.omit(PHAR)),

PARsd=sd(na.omit(PHAR)))

enviM$MOnthF<-factor(enviM$Month,levels=c(5,6,7,8,9,10,11,12,1,2,3,4))

enviM$altermonth[enviM$MOnthF=="5"]<-1

enviM$altermonth[enviM$MOnthF=="6"]<-2

enviM$altermonth[enviM$MOnthF=="7"]<-3

enviM$altermonth[enviM$MOnthF=="8"]<-4

enviM$altermonth[enviM$MOnthF=="9"]<-5

enviM$altermonth[enviM$MOnthF=="10"]<-6

enviM$altermonth[enviM$MOnthF=="11"]<-7

enviM$altermonth[enviM$MOnthF=="12"]<-8

enviM$altermonth[enviM$MOnthF=="1"]<-9

enviM$altermonth[enviM$MOnthF=="2"]<-10

enviM$altermonth[enviM$MOnthF=="3"]<-11

enviM$altermonth[enviM$MOnthF=="4"]<-12

envi$altermonth[envi$Month=="5"]<-1

envi$altermonth[envi$Month=="6"]<-2

envi$altermonth[envi$Month=="7"]<-3

envi$altermonth[envi$Month=="8"]<-4

envi$altermonth[envi$Month=="9"]<-5

envi$altermonth[envi$Month=="10"]<-6

envi$altermonth[envi$Month=="11"]<-7

envi$altermonth[envi$Month=="12"]<-8

envi$altermonth[envi$Month=="1"]<-9

envi$altermonth[envi$Month=="2"]<-10

envi$altermonth[envi$Month=="3"]<-11

envi$altermonth[envi$Month=="4"]<-12

ggplot()+geom_boxplot(data=envi,aes(x=as.factor(altermonth),y=CHLA))+

stat_smooth(data=envi,aes(x=as.numeric(altermonth),y=CHLA),

span=0.9,level=0.75,colour="green3",linetype="solid",size=1)+

theme_bw()+th+

ylab("mg/m-3")+

ggtitle(label="a. Chlorophyll-a concentration")+

xlab("Month")+ylim(0.1,0.6)

chlpm<-ggplot(enviM,aes(MOnthF,CHL))+

geom_errorbar(aes(ymin=CHL-CHLsd,ymax=CHL+CHLsd),colour="green3",linetype="solid",size=1)+

geom_point(size=3,colour="green3",shape="circle")+theme_bw()+th+

xlab("Date")+ylab("mg/m-3")+

ggtitle(label="a. Chlorophyll-a concentration")+

xlab("Month")+ylim(0.1,0.6)

chlpm

chlpm2<-ggplot(na.omit(enviM),aes(altermonth,CHL))+

stat_smooth(span=0.9,level=0.75,colour="green3",linetype="solid",size=1)+

xlab("Date")+ylab("mg/m-3")+

ggtitle(label="a. Chlorophyll-a concentration")+

xlab("Month")+ylim(0.1,0.6)+xlim(1,12)+theme_bw()+th

chlpm2

sicpm<-ggplot(enviM,aes(MOnthF,SIC))+

geom_errorbar(aes(ymin=SIC-SICsd,ymax=SIC+SICsd),colour="blue2",linetype="dashed",size=1)+

geom_point(size=3,colour="blue2",shape="triangle")+theme_bw()+th+

xlab("Date")+ylab("Fraction of tile")+ylim(0.15,0.45)+

ggtitle(label="c. Sea ice cover")+

xlab("Month")

sicpm

sicpm2<-ggplot(enviM,aes(altermonth,SIC))+

geom_ribbon(aes(ymin=SIC-SICsd,ymax=SIC+SICsd))+

xlab("Date")+ylab("mg/m-3")+ylim(0.15,0.45)+

theme_bw()+th

sicpm2

wspm<-ggplot(enviM,aes(MOnthF,WS))+

geom_errorbar(aes(ymin=WS-WSsd,ymax=WS+WSsd),colour="grey50",linetype="dotdash",size=1)+

geom_point(size=3,colour="grey50",shape="square")+theme_bw()+th+

xlab("Date")+ylab("m/sd")+

ggtitle(label="d. Surface Wind Speed")+ylim(6.5,11)+

xlab("Month")

wspm

wspm2<-ggplot(enviM,aes(altermonth,WS))+

stat_smooth(span=0.5,level=0.95,colour="grey50",linetype="dotdash",size=1)+

xlab("Date")+ylab("mg/m-3")+ylim(6.5,11)+

theme_bw()+th

wspm2

parpm<-ggplot(enviM,aes(MOnthF,PAR))+

geom_errorbar(aes(ymin=PAR-PARsd*3,ymax=PAR+PARsd*3),colour="orange3",linetype="dotted",size=1)+

geom_point(size=3,colour="orange3",shape="diamond")+theme_bw()+th+

xlab("Date")+ylab("Einstein/m2/day")+

ggtitle(label="b. Photossintetically available radiation")+ylim(-0.2,45)+

xlab("Month")

parpm

parpm2<-ggplot(enviM,aes(altermonth,PAR))+

stat_smooth(span=0.5,level=0.75,colour="orange3",linetype="dotted",size=1)+

xlab("Date")+ylab("mg/m-3")+ylim(-0.2,45)+

theme_bw()+th

parpm2

chlpm/parpm/sicpm/wspm

chlts<-ts(envi$CHLA)

sicts<-ts(envi$FRSIC)

sstts<-ts(envi$SSTemp)

wsts<-ts(envi$Wspeed)

cfts<-ts(envi$CLD)

parts<-ts(envi$PHAR)

dynlm1<-dynlm(chlts~L(sicts,0:6)+L(wsts,0:6)+

L(parts,0:6))

summary(dynlm1)

coefs<-data.frame(summary(dynlm1)$coefficients)

coefs$lag<-(c(-1:6,0:6,0:6))

head(coefs)

coefs<-subset(coefs,lag>(-1))

coefs$sig[coefs$Pr...t..<=0.05]<-"P<0.05"

coefs$sig[coefs$Pr...t..>0.05]<-"n.sig."

coefs$se<-coefs$Std..Error

coefs$vars<-c("SIC","SIC","SIC","SIC","SIC","SIC","SIC",

"WS","WS","WS","WS","WS","WS","WS",

"PAR","PAR","PAR","PAR","PAR","PAR","PAR")

parp<-ggplot()+

geom_hline(yintercept = 0,linetype="dotted",colour="grey50")+

geom_errorbar(data=subset(coefs,vars=="PAR"),

aes(x=lag,y=Estimate,ymin=Estimate-se,ymax=Estimate+se,colour=sig,

linetype=sig))+

geom_point(data=subset(coefs,vars=="PAR"),

aes(x=lag,y=Estimate,colour=sig,

shape=sig),size=3)+

theme_bw()+th+

xlab("Time lag (months)")+

labs(title="e. PAR")+

scale_colour_manual(values=c("blue","red"))

parp

sicp<-ggplot()+

geom_hline(yintercept = 0,linetype="dotted",colour="grey50")+

geom_errorbar(data=subset(coefs,vars=="SIC"),

aes(x=lag,y=Estimate,ymin=Estimate-se,ymax=Estimate+se,colour=sig,

linetype=sig))+

geom_point(data=subset(coefs,vars=="SIC"),

aes(x=lag,y=Estimate,colour=sig,

shape=sig),size=3)+

theme_bw()+th+

xlab("Time lag (months)")+

labs(title="f. SIC")+

scale_colour_manual(values=c("blue","red"))

windp<-ggplot()+

geom_hline(yintercept = 0,linetype="dotted",colour="grey50")+

geom_errorbar(data=subset(coefs,vars=="WS"),

aes(x=lag,y=Estimate,ymin=Estimate-se,ymax=Estimate+se,colour=sig,

linetype=sig))+

geom_point(data=subset(coefs,vars=="WS"),

aes(x=lag,y=Estimate,colour=sig,

shape=sig),size=3)+

theme_bw()+th+

xlab("Time lag (months)")+

labs(title="g. SWS")+

scale_colour_manual(values=c("blue","red"))

parp+sicp+windp

###---------- comparing years of sampling---------

# load shapefile polygon to sample envivars

# it is a 75km buffer around the breeding colony

shp<-shapefile("D:/DataPinguinos/Nuria/Data/Shapefile/sampleCORRECT_maskl.shp")

plot(shp)

setwd("D:/DataPinguinos/Nuria/Data/")

###-------CHL-------------

chl1920<-raster("ENVI_CHL/GIOVANNI-g4.timeAvgMap.MODISA_L3m_CHL_2018_chlor_a.20191101-20200131.65W_66S_55W_60S.tif")

chl2122<-raster("ENVI_CHL/GIOVANNI-g4.timeAvgMap.MODISA_L3m_CHL_2018_chlor_a.20211101-20220131.65W_66S_55W_60S.tif")

st<-stack(chl1920,chl2122)

chlext<-raster::extract(st, shp, method="bilinear", weights=FALSE,buffer=NULL, small=FALSE, cellnumbers=FALSE,

fun=mean, na.rm=TRUE, df=TRUE, factors=FALSE,sp=TRUE)

names(chlext)[names(chlext) == 'GIOVANNI.g4.timeAvgMap.MODISA_L3m_CHL_2018_chlor_a.20191101.20200131.65W_66S_55W_60S'] <- 'CHL1920'

names(chlext)[names(chlext) == 'GIOVANNI.g4.timeAvgMap.MODISA_L3m_CHL_2018_chlor_a.20211101.20220131.65W_66S_55W_60S'] <- 'CHL2122'

head(chlext)

chldf<-data.frame(chlext)

head(chldf)

chlM<-melt(chldf[2:4],id.vars=c("ColDist"))

### ----------PAR--------------

par1920<-raster("ENVI_PAR/GIOVANNI-g4.timeAvgMap.MODISA_L3m_PAR_2018_par.20191101-20200131.65W_66S_55W_60S.tif")

par2122<-raster("ENVI_PAR/GIOVANNI-g4.timeAvgMap.MODISA_L3m_PAR_2018_par.20211101-20220131.65W_66S_55W_60S.tif")

st.par<-stack(par1920,par2122)

parext<-raster::extract(st.par, shp, method="bilinear", weights=FALSE,buffer=NULL, small=FALSE, cellnumbers=FALSE,

fun=mean, na.rm=TRUE, df=TRUE, factors=FALSE,sp=TRUE)

head(parext)

names(parext)[names(parext) == 'GIOVANNI.g4.timeAvgMap.MODISA_L3m_PAR_2018_par.20191101.20200131.65W_66S_55W_60S'] <- 'PAR1920'

names(parext)[names(parext) == 'GIOVANNI.g4.timeAvgMap.MODISA_L3m_PAR_2018_par.20211101.20220131.65W_66S_55W_60S'] <- 'PAR2122'

pardf<-data.frame(parext)

parM<-melt(pardf[2:4],id.vars=c("ColDist"))

head(parM)

#---------SIC--------------------------

sic19<-raster("ENVI_SIC/GIOVANNI-g4.timeAvgMap.M2TMNXFLX_5_12_4_FRSEAICE.20190601-20190930.65W_66S_55W_60S.tif")

sic21<-raster("ENVI_SIC/GIOVANNI-g4.timeAvgMap.M2TMNXFLX_5_12_4_FRSEAICE.20210601-20210930.65W_66S_55W_60S.tif")

st.sic<-stack(sic19,sic21)

sicext<-raster::extract(st.sic, shp, method="bilinear", weights=FALSE,buffer=NULL, small=FALSE, cellnumbers=FALSE,

fun=mean, na.rm=TRUE, df=TRUE, factors=FALSE,sp=TRUE)

names(sicext)[names(sicext) == 'GIOVANNI.g4.timeAvgMap.M2TMNXFLX_5_12_4_FRSEAICE.20190601.20190930.65W_66S_55W_60S'] <- 'SIC19'

names(sicext)[names(sicext) == 'GIOVANNI.g4.timeAvgMap.M2TMNXFLX_5_12_4_FRSEAICE.20210601.20210930.65W_66S_55W_60S'] <- 'SIC21'

sicdf<-data.frame(sicext)

sicM<-melt(sicdf[2:4],id.vars=c("ColDist"))

###-------SWS--------------------------

ws1920<-raster("ENVI_SWS/GIOVANNI-g4.timeAvgMap.M2TMNXFLX_5_12_4_SPEED.20191101-20200131.65W_66S_55W_60S.tif")

ws2122<-raster("ENVI_SWS/GIOVANNI-g4.timeAvgMap.M2TMNXFLX_5_12_4_SPEED.20211101-20220131.65W_66S_55W_60S.tif")

st.ws<-stack(ws1920,ws2122)

wsext<-raster::extract(st.ws, shp, method="bilinear", weights=FALSE,buffer=NULL, small=FALSE, cellnumbers=FALSE,

fun=mean, na.rm=TRUE, df=TRUE, factors=FALSE,sp=TRUE)

names(wsext)[names(wsext) == 'GIOVANNI.g4.timeAvgMap.M2TMNXFLX_5_12_4_SPEED.20191101.20200131.65W_66S_55W_60S'] <- 'SWS1920'

names(wsext)[names(wsext) == 'GIOVANNI.g4.timeAvgMap.M2TMNXFLX_5_12_4_SPEED.20211101.20220131.65W_66S_55W_60S'] <- 'SWS2122'

wsdf<-data.frame(wsext)

wsM<-melt(wsdf[2:4],id.vars=c("ColDist"))

#### ----------------- Krill density analysis----------------------------

library(sf)

library(maptools)

library(raster)

library(spatstat)

library(plyr)

?Kest

krill22<-read.csv("D:/DataPinguinos/Nuria/Data/KrillAcoustic/01-2022.csv")

krill20<-read.csv("D:/DataPinguinos/Nuria/Data/KrillAcoustic/01_2020.csv")

krill19<-read.csv("D:/DataPinguinos/Nuria/Data/KrillAcoustic/12-2019.csv")

head(krill22)

summary(krill22$PRC_NASC)

krill22$PRC_NASC[krill22$PRC_NASC<0]<-0

krill22$PRC_NASC[krill22$PRC_NASC>13000]<-0

krill20$PRC_NASC[krill20$PRC_NASC<0]<-0

krill19$PRC_NASC[krill19$PRC_NASC<0]<-0

k22<-data.frame(season=c("2021/22"),date=c("Jan2022"),grid=krill22$Interval,

depth.strata=krill22$Layer_depth_min,depthMean=krill22$Depth_mean, NASC=krill22$PRC_NASC,

Lon=krill22$Lon_M,Lat=krill22$Lat_M)

k20<-data.frame(season=c("2019/20"),date=c("Jan2020"),grid=krill20$Interval,

depth.strata=krill20$Layer_depth_min,depthMean=krill20$Depth_mean, NASC=krill20$PRC_NASC,

Lon=krill20$Lon_M,Lat=krill20$Lat_M)

k19<-data.frame(season=c("2019/20"),date=c("Dec2019"),grid=krill19$Interval,

depth.strata=krill19$Layer_depth_min,depthMean=krill19$Depth_mean, NASC=krill19$PRC_NASC,

Lon=krill19$Lon_M,Lat=krill19$Lat_M)

krill19$year<-substring(krill19$Date_S,first=1,last=4)

krill19$month<-substring(krill19$Date_S,first=5,last=6)

krill19$day<-substring(krill19$Date_S,first=7,last=8)

krill19$date<-paste(krill19$year,krill19$month,krill19$day,sep="-")

krill19$TS<-as.POSIXct(strptime(paste(krill19$date,krill19$Time_S), format="%Y-%m-%d %H:%M:%S", tz="GMT"))

### ------------ mean krill density-------------------

k19m<-ddply(k19, c("season","date","grid","depth.strata"), summarise,

depthM=mean(depthMean),

NASCs=sum(NASC),

NASCm=mean(NASC),

Lon=mean(na.omit(Lon)),

Lat=mean(na.omit(Lat)))

k20m<-ddply(k20, c("season","date","grid","depth.strata"), summarise,

depthM=mean(depthMean),

NASCs=sum(NASC),

NASCm=mean(NASC),

Lon=mean(na.omit(Lon)),

Lat=mean(na.omit(Lat)))

k22m<-ddply(k22, c("season","date","grid","depth.strata"), summarise,

depthM=mean(depthMean),

NASCs=sum(NASC),

NASCm=mean(NASC),

Lon=mean(na.omit(Lon)),

Lat=mean(na.omit(Lat)))

kall<-rbind(k19m,k20m,k22m)

kallm<-ddply(kall, c("season","depth.strata"), summarise,

mean=mean(NASCm),

sum=sum(NASCs),

sd=sd(NASCm),

se=sd/sqrt(length(NASCm)),

median=median(NASCm[NASCm>0]),

min=min(NASCm[NASCm>0]),

max=max(NASCm),

Lon=mean(na.omit(Lon)),

Lat=mean(na.omit(Lat)))

kallm2<-ddply(kall, c("season","depth.strata"), summarise,

mean=mean(NASCm[NASCm>0]),

sum=sum(NASCs),

sd=sd(NASCm[NASCm>0]),

se=sd/sqrt(length(NASCm[NASCm>0])),

median=median(NASCm[NASCm>0]),

min=min(NASCm[NASCm>0]),

max=max(NASCm),

Lon=mean(na.omit(Lon)),

Lat=mean(na.omit(Lat)))

q90=quantile(kallm2$sum,probs=0.9)

q80=quantile(kallm2$sum,probs=0.8)

kallm$min[kall$min=="inf"]<-0.00001

ggplot((kallm), aes(-1*depth.strata,mean,colour=season,linetype=season,shape=season))+

geom_vline(xintercept=-87,colour="red",

linetype="dashed")+

geom_vline(xintercept=-50,colour="blue",

linetype="dotted")+

stat_smooth(span=0.25)+

geom_errorbar(aes(ymin=mean-se,ymax=mean+se))+

geom_point()+

xlab("Mean krill density")+

coord_flip()+theme_bw()+th+xlim(-300,0)+

scale_colour_manual(values=c("blue","red"))+

scale_linetype_manual(values=c("dashed","dotted"))

ggplot(kallm2, aes(-1*depth.strata,sum,colour=season,linetype=season,shape=season))+

geom_vline(xintercept=-87,colour="red",

linetype="dashed")+

geom_errorbar(aes(ymin=sum-q80,ymax=sum+q80))+

geom_vline(xintercept=-50,colour="blue",

linetype="dotted")+

stat_smooth(span=0.25)+

geom_point()+

xlab("Accumulated krill density")+

coord_flip()+theme_bw()+th+xlim(-300,0)+

scale_colour_manual(values=c("blue","red"))+

scale_linetype_manual(values=c("dashed","dotted"))

###permanova

head(kall)

kallm3<-ddply(kall, c("season","date","depth.strata"), summarise,

mean=mean(NASCm),

sum=sum(NASCs),

sd=sd(NASCm),

se=sd/sqrt(length(NASCm)),

median=median(NASCm[NASCm>0]),

min=min(NASCm[NASCm>0]),

max=max(NASCm),

Lon=mean(na.omit(Lon)),

Lat=mean(na.omit(Lat)))

library(PERMANOVA)

Xtd=IniTransform(as.matrix(kallm3$sum),

transform="Standardize columns")

TD=DistContinuous(Xtd)

ftd<-PERMANOVA(TD,group=as.factor(kallm3$season),nperm=999)

print(ftd)$Initial$Global

summary(ftd)

kallm3b<-subset(kallm3,mean>10)

Xtd2=IniTransform(as.matrix(kallm3b$depth.strata),

transform="Standardize columns")

TD2=DistContinuous(Xtd2)

ftd2<-PERMANOVA(TD2,as.factor(kallm3b$season),nperm=999)

print(ftd2)$Initial$Global

summary(ftd2)

summary(as.factor(k19m$depth.strata))

#### --------------spatial aggregation-------------

head(kall)

##---------2019----------------------------

#### separate each depth strata with detcetd krill swarms

st1<-subset(k19,depth.strata=="0"& NASC>0)

st2<-subset(k19,depth.strata=="5"& NASC>0)

st3<-subset(k19,depth.strata=="10"& NASC>0)

st4<-subset(k19,depth.strata=="15"& NASC>0)

st5<-subset(k19,depth.strata=="20"& NASC>0)

st6<-subset(k19,depth.strata=="25"& NASC>0)

st7<-subset(k19,depth.strata=="30"& NASC>0)

st8<-subset(k19,depth.strata=="35"& NASC>0)

st9<-subset(k19,depth.strata=="40"& NASC>0)

st10<-subset(k19,depth.strata=="45"& NASC>0)

st11<-subset(k19,depth.strata=="50"& NASC>0)

st12<-subset(k19,depth.strata=="55"& NASC>0)

st13<-subset(k19,depth.strata=="60"& NASC>0)

st14<-subset(k19,depth.strata=="65"& NASC>0)

st15<-subset(k19,depth.strata=="70"& NASC>0)

st16<-subset(k19,depth.strata=="75"& NASC>0)

st17<-subset(k19,depth.strata=="80"& NASC>0)

st18<-subset(k19,depth.strata=="85"& NASC>0)

st19<-subset(k19,depth.strata=="90"& NASC>0)

st20<-subset(k19,depth.strata=="95"& NASC>0)

st21<-subset(k19,depth.strata=="100"& NASC>0)

st22<-subset(k19,depth.strata=="105"& NASC>0)

st23<-subset(k19,depth.strata=="110"& NASC>0)

st24<-subset(k19,depth.strata=="115"& NASC>0)

st25<-subset(k19,depth.strata=="120"& NASC>0)

st26<-subset(k19,depth.strata=="125"& NASC>0)

st27<-subset(k19,depth.strata=="130"& NASC>0)

st28<-subset(k19,depth.strata=="135"& NASC>0)

st29<-subset(k19,depth.strata=="140"& NASC>0)

st30<-subset(k19,depth.strata=="145"& NASC>0)

st31<-subset(k19,depth.strata=="150"& NASC>0)

st32<-subset(k19,depth.strata=="155"& NASC>0)

st33<-subset(k19,depth.strata=="160"& NASC>0)

st34<-subset(k19,depth.strata=="165"& NASC>0)

st35<-subset(k19,depth.strata=="170"& NASC>0)

st36<-subset(k19,depth.strata=="175"& NASC>0)

st37<-subset(k19,depth.strata=="180"& NASC>0)

st38<-subset(k19,depth.strata=="185"& NASC>0)

st39<-subset(k19,depth.strata=="190"& NASC>0)

st40<-subset(k19,depth.strata=="195"& NASC>0)

st41<-subset(k19,depth.strata=="200"& NASC>0)

st42<-subset(k19,depth.strata=="205"& NASC>0)

st43<-subset(k19,depth.strata=="210"& NASC>0)

st44<-subset(k19,depth.strata=="215"& NASC>0)

st45<-subset(k19,depth.strata=="220"& NASC>0)

st46<-subset(k19,depth.strata=="225"& NASC>0)

st47<-subset(k19,depth.strata=="230"& NASC>0)

st48<-subset(k19,depth.strata=="235"& NASC>0)

st49<-subset(k19,depth.strata=="240"& NASC>0)

st50<-subset(k19,depth.strata=="245"& NASC>0)

st51<-subset(k19,depth.strata=="250"& NASC>0)

st52<-subset(k19,depth.strata=="255"& NASC>0)

st53<-subset(k19,depth.strata=="260"& NASC>0)

st54<-subset(k19,depth.strata=="265"& NASC>0)

st55<-subset(k19,depth.strata=="270"& NASC>0)

st56<-subset(k19,depth.strata=="275"& NASC>0)

st57<-subset(k19,depth.strata=="280"& NASC>0)

st58<-subset(k19,depth.strata=="285"& NASC>0)

st59<-subset(k19,depth.strata=="290"& NASC>0)

st60<-subset(k19,depth.strata=="295"& NASC>0)

st61<-subset(k19,depth.strata=="300"& NASC>0)

head(st61)

### create a spatial points data frame for each

spdf1<-as.ppp(SpatialPointsDataFrame(coords=coordinates(st1[7:8]),st1))

spdf2<-as.ppp(SpatialPointsDataFrame(coords=coordinates(st2[7:8]),st2))

spdf3<-as.ppp(SpatialPointsDataFrame(coords=coordinates(st3[7:8]),st3))

spdf4<-as.ppp(SpatialPointsDataFrame(coords=coordinates(st4[7:8]),st4))

spdf5<-as.ppp(SpatialPointsDataFrame(coords=coordinates(st5[7:8]),st5))

spdf6<-as.ppp(SpatialPointsDataFrame(coords=coordinates(st6[7:8]),st6))

spdf7<-as.ppp(SpatialPointsDataFrame(coords=coordinates(st7[7:8]),st7))

spdf8<-as.ppp(SpatialPointsDataFrame(coords=coordinates(st8[7:8]),st8))

spdf9<-as.ppp(SpatialPointsDataFrame(coords=coordinates(st9[7:8]),st9))

spdf10<-as.ppp(SpatialPointsDataFrame(coords=coordinates(st10[7:8]),st10))

spdf11<-as.ppp(SpatialPointsDataFrame(coords=coordinates(st11[7:8]),st11))

spdf12<-as.ppp(SpatialPointsDataFrame(coords=coordinates(st12[7:8]),st12))

spdf13<-as.ppp(SpatialPointsDataFrame(coords=coordinates(st13[7:8]),st13))

spdf14<-as.ppp(SpatialPointsDataFrame(coords=coordinates(st14[7:8]),st14))

spdf15<-as.ppp(SpatialPointsDataFrame(coords=coordinates(st15[7:8]),st15))

spdf16<-as.ppp(SpatialPointsDataFrame(coords=coordinates(st16[7:8]),st16))

spdf17<-as.ppp(SpatialPointsDataFrame(coords=coordinates(st17[7:8]),st17))

spdf18<-as.ppp(SpatialPointsDataFrame(coords=coordinates(st18[7:8]),st18))

spdf19<-as.ppp(SpatialPointsDataFrame(coords=coordinates(st19[7:8]),st19))

spdf20<-as.ppp(SpatialPointsDataFrame(coords=coordinates(st20[7:8]),st20))

spdf21<-as.ppp(SpatialPointsDataFrame(coords=coordinates(st21[7:8]),st21))

spdf22<-as.ppp(SpatialPointsDataFrame(coords=coordinates(st22[7:8]),st22))

spdf23<-as.ppp(SpatialPointsDataFrame(coords=coordinates(st23[7:8]),st23))

spdf24<-as.ppp(SpatialPointsDataFrame(coords=coordinates(st24[7:8]),st24))

spdf25<-as.ppp(SpatialPointsDataFrame(coords=coordinates(st25[7:8]),st25))

spdf26<-as.ppp(SpatialPointsDataFrame(coords=coordinates(st26[7:8]),st26))

spdf27<-as.ppp(SpatialPointsDataFrame(coords=coordinates(st27[7:8]),st27))

spdf28<-as.ppp(SpatialPointsDataFrame(coords=coordinates(st28[7:8]),st28))

spdf29<-as.ppp(SpatialPointsDataFrame(coords=coordinates(st29[7:8]),st29))

spdf30<-as.ppp(SpatialPointsDataFrame(coords=coordinates(st30[7:8]),st30))

spdf31<-as.ppp(SpatialPointsDataFrame(coords=coordinates(st31[7:8]),st31))

spdf32<-as.ppp(SpatialPointsDataFrame(coords=coordinates(st32[7:8]),st32))

spdf33<-as.ppp(SpatialPointsDataFrame(coords=coordinates(st33[7:8]),st33))

spdf34<-as.ppp(SpatialPointsDataFrame(coords=coordinates(st34[7:8]),st34))

spdf35<-as.ppp(SpatialPointsDataFrame(coords=coordinates(st35[7:8]),st35))

spdf36<-as.ppp(SpatialPointsDataFrame(coords=coordinates(st36[7:8]),st36))

spdf37<-as.ppp(SpatialPointsDataFrame(coords=coordinates(st37[7:8]),st37))

spdf38<-as.ppp(SpatialPointsDataFrame(coords=coordinates(st38[7:8]),st38))

spdf39<-as.ppp(SpatialPointsDataFrame(coords=coordinates(st39[7:8]),st39))

spdf40<-as.ppp(SpatialPointsDataFrame(coords=coordinates(st40[7:8]),st40))

spdf41<-as.ppp(SpatialPointsDataFrame(coords=coordinates(st41[7:8]),st41))

spdf42<-as.ppp(SpatialPointsDataFrame(coords=coordinates(st42[7:8]),st42))

spdf43<-as.ppp(SpatialPointsDataFrame(coords=coordinates(st43[7:8]),st43))

spdf44<-as.ppp(SpatialPointsDataFrame(coords=coordinates(st44[7:8]),st44))

spdf45<-as.ppp(SpatialPointsDataFrame(coords=coordinates(st45[7:8]),st45))

spdf46<-as.ppp(SpatialPointsDataFrame(coords=coordinates(st46[7:8]),st46))

spdf47<-as.ppp(SpatialPointsDataFrame(coords=coordinates(st47[7:8]),st47))

spdf48<-as.ppp(SpatialPointsDataFrame(coords=coordinates(st48[7:8]),st48))

spdf49<-as.ppp(SpatialPointsDataFrame(coords=coordinates(st49[7:8]),st49))

spdf50<-as.ppp(SpatialPointsDataFrame(coords=coordinates(st50[7:8]),st50))

spdf51<-as.ppp(SpatialPointsDataFrame(coords=coordinates(st51[7:8]),st51))

spdf52<-as.ppp(SpatialPointsDataFrame(coords=coordinates(st52[7:8]),st52))

spdf53<-as.ppp(SpatialPointsDataFrame(coords=coordinates(st53[7:8]),st53))

spdf54<-as.ppp(SpatialPointsDataFrame(coords=coordinates(st54[7:8]),st54))

spdf55<-as.ppp(SpatialPointsDataFrame(coords=coordinates(st55[7:8]),st55))

spdf56<-as.ppp(SpatialPointsDataFrame(coords=coordinates(st56[7:8]),st56))

spdf57<-as.ppp(SpatialPointsDataFrame(coords=coordinates(st57[7:8]),st57))

spdf58<-as.ppp(SpatialPointsDataFrame(coords=coordinates(st58[7:8]),st58))

spdf59<-as.ppp(SpatialPointsDataFrame(coords=coordinates(st59[7:8]),st59))

spdf60<-as.ppp(SpatialPointsDataFrame(coords=coordinates(st60[7:8]),st60))

spdf61<-as.ppp(SpatialPointsDataFrame(coords=coordinates(st61[7:8]),st61))

kest1<-Kest(spdf1,correction="Ripley")

kest2<-Kest(spdf2,correction="Ripley")

kest3<-Kest(spdf3,correction="Ripley")

kest4<-Kest(spdf4,correction="Ripley")

kest5<-Kest(spdf5,correction="Ripley")

kest6<-Kest(spdf6,correction="Ripley")

kest7<-Kest(spdf7,correction="Ripley")

kest8<-Kest(spdf8,correction="Ripley")

kest9<-Kest(spdf9,correction="Ripley")

kest10<-Kest(spdf10,correction="Ripley")

kest11<-Kest(spdf11,correction="Ripley")

kest12<-Kest(spdf12,correction="Ripley")

kest13<-Kest(spdf13,correction="Ripley")

kest14<-Kest(spdf14,correction="Ripley")

kest15<-Kest(spdf15,correction="Ripley")

kest16<-Kest(spdf16,correction="Ripley")

kest17<-Kest(spdf17,correction="Ripley")

kest18<-Kest(spdf18,correction="Ripley")

kest19<-Kest(spdf19,correction="Ripley")

kest20<-Kest(spdf20,correction="Ripley")

kest21<-Kest(spdf21,correction="Ripley")

kest22<-Kest(spdf22,correction="Ripley")

kest23<-Kest(spdf23,correction="Ripley")

kest24<-Kest(spdf24,correction="Ripley")

kest25<-Kest(spdf25,correction="Ripley")

kest26<-Kest(spdf26,correction="Ripley")

kest27<-Kest(spdf27,correction="Ripley")

kest28<-Kest(spdf28,correction="Ripley")

kest29<-Kest(spdf29,correction="Ripley")

kest30<-Kest(spdf30,correction="Ripley")

kest31<-Kest(spdf31,correction="Ripley")

kest32<-Kest(spdf32,correction="Ripley")

kest33<-Kest(spdf33,correction="Ripley")

kest34<-Kest(spdf34,correction="Ripley")

kest35<-Kest(spdf35,correction="Ripley")

kest36<-Kest(spdf36,correction="Ripley")

kest37<-Kest(spdf37,correction="Ripley")

kest38<-Kest(spdf38,correction="Ripley")

kest39<-Kest(spdf39,correction="Ripley")

kest40<-Kest(spdf40,correction="Ripley")

kest41<-Kest(spdf41,correction="Ripley")

kest42<-Kest(spdf42,correction="Ripley")

kest43<-Kest(spdf43,correction="Ripley")

st1.19m<-mean(kest1$iso-kest1$theo)

st2.19m<-mean(kest2$iso-kest2$theo)

st3.19m<-mean(kest3$iso-kest3$theo)

st4.19m<-mean(kest4$iso-kest4$theo)

st5.19m<-mean(kest5$iso-kest5$theo)

st6.19m<-mean(kest6$iso-kest6$theo)

st7.19m<-mean(kest7$iso-kest7$theo)

st8.19m<-mean(kest8$iso-kest8$theo)

st9.19m<-mean(kest9$iso-kest9$theo)

st10.19m<-mean(kest10$iso-kest10$theo)

st11.19m<-mean(kest11$iso-kest11$theo)

st12.19m<-mean(kest12$iso-kest12$theo)

st13.19m<-mean(kest13$iso-kest13$theo)

st14.19m<-mean(kest14$iso-kest14$theo)

st15.19m<-mean(kest15$iso-kest15$theo)

st16.19m<-mean(kest16$iso-kest16$theo)

st17.19m<-mean(kest17$iso-kest17$theo)

st18.19m<-mean(kest18$iso-kest18$theo)

st19.19m<-mean(kest19$iso-kest19$theo)

st20.19m<-mean(kest20$iso-kest20$theo)

st21.19m<-mean(kest21$iso-kest21$theo)

st22.19m<-mean(kest22$iso-kest22$theo)

st23.19m<-mean(kest23$iso-kest23$theo)

st24.19m<-mean(kest24$iso-kest24$theo)

st25.19m<-mean(kest25$iso-kest25$theo)

st26.19m<-mean(kest26$iso-kest26$theo)

st27.19m<-mean(kest27$iso-kest27$theo)

st28.19m<-mean(kest28$iso-kest28$theo)

st29.19m<-mean(kest29$iso-kest29$theo)

st30.19m<-mean(kest30$iso-kest30$theo)

st31.19m<-mean(kest31$iso-kest31$theo)

st32.19m<-mean(kest32$iso-kest32$theo)

st33.19m<-mean(kest33$iso-kest33$theo)

st34.19m<-mean(kest34$iso-kest34$theo)

st35.19m<-mean(kest35$iso-kest35$theo)

st36.19m<-mean(kest36$iso-kest36$theo)

st37.19m<-mean(kest37$iso-kest37$theo)

st38.19m<-mean(kest38$iso-kest38$theo)

st39.19m<-mean(kest39$iso-kest39$theo)

st40.19m<-mean(kest40$iso-kest40$theo)

st41.19m<-mean(kest41$iso-kest41$theo)

st42.19m<-mean(kest42$iso-kest42$theo)

st43.19m<-mean(kest43$iso-kest43$theo)

st44.19m<-mean(kest44$iso-kest44$theo)

st45.19m<-mean(kest45$iso-kest45$theo)

st46.19m<-mean(kest46$iso-kest46$theo)

st47.19m<-mean(kest47$iso-kest47$theo)

st48.19m<-mean(kest48$iso-kest48$theo)

st49.19m<-mean(kest49$iso-kest49$theo)

st50.19m<-mean(kest50$iso-kest50$theo)

st51.19m<-mean(kest51$iso-kest51$theo)

st52.19m<-mean(kest52$iso-kest52$theo)

st1.19sd<-sd(kest1$iso-kest1$theo)

st2.19sd<-sd(kest2$iso-kest2$theo)

st3.19sd<-sd(kest3$iso-kest3$theo)

st4.19sd<-sd(kest4$iso-kest4$theo)

st5.19sd<-sd(kest5$iso-kest5$theo)

st6.19sd<-sd(kest6$iso-kest6$theo)

st7.19sd<-sd(kest7$iso-kest7$theo)

st8.19sd<-sd(kest8$iso-kest8$theo)

st9.19sd<-sd(kest9$iso-kest9$theo)

st10.19sd<-sd(kest10$iso-kest10$theo)

st11.19sd<-sd(kest11$iso-kest11$theo)

st12.19sd<-sd(kest12$iso-kest12$theo)

st13.19sd<-sd(kest13$iso-kest13$theo)

st14.19sd<-sd(kest14$iso-kest14$theo)

st15.19sd<-sd(kest15$iso-kest15$theo)

st16.19sd<-sd(kest16$iso-kest16$theo)

st17.19sd<-sd(kest17$iso-kest17$theo)

st18.19sd<-sd(kest18$iso-kest18$theo)

st19.19sd<-sd(kest19$iso-kest19$theo)

st20.19sd<-sd(kest20$iso-kest20$theo)

st21.19sd<-sd(kest21$iso-kest21$theo)

st22.19sd<-sd(kest22$iso-kest22$theo)

st23.19sd<-sd(kest23$iso-kest23$theo)

st24.19sd<-sd(kest24$iso-kest24$theo)

st25.19sd<-sd(kest25$iso-kest25$theo)

st26.19sd<-sd(kest26$iso-kest26$theo)

st27.19sd<-sd(kest27$iso-kest27$theo)

st28.19sd<-sd(kest28$iso-kest28$theo)

st29.19sd<-sd(kest29$iso-kest29$theo)

st30.19sd<-sd(kest30$iso-kest30$theo)

st31.19sd<-sd(kest31$iso-kest31$theo)

st32.19sd<-sd(kest32$iso-kest32$theo)

st33.19sd<-sd(kest33$iso-kest33$theo)

st34.19sd<-sd(kest34$iso-kest34$theo)

st35.19sd<-sd(kest35$iso-kest35$theo)

st36.19sd<-sd(kest36$iso-kest36$theo)

st37.19sd<-sd(kest37$iso-kest37$theo)

st38.19sd<-sd(kest38$iso-kest38$theo)

st39.19sd<-sd(kest39$iso-kest39$theo)

st40.19sd<-sd(kest40$iso-kest40$theo)

st41.19sd<-sd(kest41$iso-kest41$theo)

st42.19sd<-sd(kest42$iso-kest42$theo)

st43.19sd<-sd(kest43$iso-kest43$theo)

st44.19sd<-sd(kest44$iso-kest44$theo)

st45.19sd<-sd(kest45$iso-kest45$theo)

st46.19sd<-sd(kest46$iso-kest46$theo)

st47.19sd<-sd(kest47$iso-kest47$theo)

st48.19sd<-sd(kest48$iso-kest48$theo)

st49.19sd<-sd(kest49$iso-kest49$theo)

st50.19sd<-sd(kest50$iso-kest50$theo)

st51.19sd<-sd(kest51$iso-kest51$theo)

st52.19sd<-sd(kest52$iso-kest52$theo)

mean19<-data.frame( mean=rbind(st1.19m,st2.19m,st3.19m,st4.19m,st5.19m,st6.19m,st7.19m,st8.19m,st9.19m,st10.19m, st11.19m,st12.19m,st13.19m,st14.19m,st15.19m,st16.19m,st17.19m,st18.19m,st19.19m,st20.19m, st21.19m,st22.19m,st23.19m,st24.19m,st25.19m,st26.19m,st27.19m,st28.19m,st29.19m,st30.19m, st31.19m,st32.19m,st33.19m,st34.19m,st35.19m,st36.19m,st37.19m,st38.19m,st39.19m,st40.19m), sd=rbind(st1.19sd,st2.19sd,st3.19sd,st4.19sd,st5.19sd,st6.19sd,st7.19sd,st8.19sd,st9.19sd,st10.19sd, st11.19sd,st12.19sd,st13.19sd,st14.19sd,st15.19sd,st16.19sd,st17.19sd,st18.19sd,st19.19sd,st20.19sd, st21.19sd,st22.19sd,st23.19sd,st24.19sd,st25.19sd,st26.19sd,st27.19sd,st28.19sd,st29.19sd,st30.19sd, st31.19sd,st32.19sd,st33.19sd,st34.19sd,st35.19sd,st36.19sd,st37.19sd,st38.19sd,st39.19sd,st40.19sd), depth=(c(1:40)*5)-5,year=c(2019),season=c("2019/20"))

###-----------2020-------------------------------------------

#### separate each depth strata with detcetd krill swarms

st1<-subset(k20,depth.strata=="0"& NASC>0)

st2<-subset(k20,depth.strata=="5"& NASC>0)

st3<-subset(k20,depth.strata=="10"& NASC>0)

st4<-subset(k20,depth.strata=="15"& NASC>0)

st5<-subset(k20,depth.strata=="20"& NASC>0)

st6<-subset(k20,depth.strata=="25"& NASC>0)

st7<-subset(k20,depth.strata=="30"& NASC>0)

st8<-subset(k20,depth.strata=="35"& NASC>0)

st9<-subset(k20,depth.strata=="40"& NASC>0)

st10<-subset(k20,depth.strata=="45"& NASC>0)

st11<-subset(k20,depth.strata=="50"& NASC>0)

st12<-subset(k20,depth.strata=="55"& NASC>0)

st13<-subset(k20,depth.strata=="60"& NASC>0)

st14<-subset(k20,depth.strata=="65"& NASC>0)

st15<-subset(k20,depth.strata=="70"& NASC>0)

st16<-subset(k20,depth.strata=="75"& NASC>0)

st17<-subset(k20,depth.strata=="80"& NASC>0)

st18<-subset(k20,depth.strata=="85"& NASC>0)

st19<-subset(k20,depth.strata=="90"& NASC>0)

st20<-subset(k20,depth.strata=="95"& NASC>0)

st21<-subset(k20,depth.strata=="100"& NASC>0)

st22<-subset(k20,depth.strata=="105"& NASC>0)

st23<-subset(k20,depth.strata=="110"& NASC>0)

st24<-subset(k20,depth.strata=="115"& NASC>0)

st25<-subset(k20,depth.strata=="120"& NASC>0)

st26<-subset(k20,depth.strata=="125"& NASC>0)

st27<-subset(k20,depth.strata=="130"& NASC>0)

st28<-subset(k20,depth.strata=="135"& NASC>0)

st29<-subset(k20,depth.strata=="140"& NASC>0)

st30<-subset(k20,depth.strata=="145"& NASC>0)

st31<-subset(k20,depth.strata=="150"& NASC>0)

st32<-subset(k20,depth.strata=="155"& NASC>0)

st33<-subset(k20,depth.strata=="160"& NASC>0)

st34<-subset(k20,depth.strata=="165"& NASC>0)

st35<-subset(k20,depth.strata=="170"& NASC>0)

st36<-subset(k20,depth.strata=="175"& NASC>0)

st37<-subset(k20,depth.strata=="180"& NASC>0)

st38<-subset(k20,depth.strata=="185"& NASC>0)

st39<-subset(k20,depth.strata=="190"& NASC>0)

st40<-subset(k20,depth.strata=="195"& NASC>0)

st41<-subset(k20,depth.strata=="200"& NASC>0)

st42<-subset(k20,depth.strata=="205"& NASC>0)

st43<-subset(k20,depth.strata=="210"& NASC>0)

st44<-subset(k20,depth.strata=="215"& NASC>0)

st45<-subset(k20,depth.strata=="220"& NASC>0)

st46<-subset(k20,depth.strata=="225"& NASC>0)

st47<-subset(k20,depth.strata=="230"& NASC>0)

st48<-subset(k20,depth.strata=="235"& NASC>0)

st49<-subset(k20,depth.strata=="240"& NASC>0)

st50<-subset(k20,depth.strata=="245"& NASC>0)

st51<-subset(k20,depth.strata=="250"& NASC>0)

st52<-subset(k20,depth.strata=="255"& NASC>0)

st53<-subset(k20,depth.strata=="260"& NASC>0)

st54<-subset(k20,depth.strata=="265"& NASC>0)

st55<-subset(k20,depth.strata=="270"& NASC>0)

st56<-subset(k20,depth.strata=="275"& NASC>0)

st57<-subset(k20,depth.strata=="280"& NASC>0)

st58<-subset(k20,depth.strata=="285"& NASC>0)

st59<-subset(k20,depth.strata=="290"& NASC>0)

st60<-subset(k20,depth.strata=="295"& NASC>0)

st61<-subset(k20,depth.strata=="300"& NASC>0)

### create a spatial points data frame for each

spdf1<-as.ppp(SpatialPointsDataFrame(coords=coordinates(st1[7:8]),st1))

spdf2<-as.ppp(SpatialPointsDataFrame(coords=coordinates(st2[7:8]),st2))

spdf3<-as.ppp(SpatialPointsDataFrame(coords=coordinates(st3[7:8]),st3))

spdf4<-as.ppp(SpatialPointsDataFrame(coords=coordinates(st4[7:8]),st4))

spdf5<-as.ppp(SpatialPointsDataFrame(coords=coordinates(st5[7:8]),st5))

spdf6<-as.ppp(SpatialPointsDataFrame(coords=coordinates(st6[7:8]),st6))

spdf7<-as.ppp(SpatialPointsDataFrame(coords=coordinates(st7[7:8]),st7))

spdf8<-as.ppp(SpatialPointsDataFrame(coords=coordinates(st8[7:8]),st8))

spdf9<-as.ppp(SpatialPointsDataFrame(coords=coordinates(st9[7:8]),st9))

spdf10<-as.ppp(SpatialPointsDataFrame(coords=coordinates(st10[7:8]),st10))

spdf11<-as.ppp(SpatialPointsDataFrame(coords=coordinates(st11[7:8]),st11))

spdf12<-as.ppp(SpatialPointsDataFrame(coords=coordinates(st12[7:8]),st12))

spdf13<-as.ppp(SpatialPointsDataFrame(coords=coordinates(st13[7:8]),st13))

spdf14<-as.ppp(SpatialPointsDataFrame(coords=coordinates(st14[7:8]),st14))

spdf15<-as.ppp(SpatialPointsDataFrame(coords=coordinates(st15[7:8]),st15))

spdf16<-as.ppp(SpatialPointsDataFrame(coords=coordinates(st16[7:8]),st16))

spdf17<-as.ppp(SpatialPointsDataFrame(coords=coordinates(st17[7:8]),st17))

spdf18<-as.ppp(SpatialPointsDataFrame(coords=coordinates(st18[7:8]),st18))

spdf19<-as.ppp(SpatialPointsDataFrame(coords=coordinates(st19[7:8]),st19))

spdf20<-as.ppp(SpatialPointsDataFrame(coords=coordinates(st20[7:8]),st20))

spdf21<-as.ppp(SpatialPointsDataFrame(coords=coordinates(st21[7:8]),st21))

spdf22<-as.ppp(SpatialPointsDataFrame(coords=coordinates(st22[7:8]),st22))

spdf23<-as.ppp(SpatialPointsDataFrame(coords=coordinates(st23[7:8]),st23))

spdf24<-as.ppp(SpatialPointsDataFrame(coords=coordinates(st24[7:8]),st24))

spdf25<-as.ppp(SpatialPointsDataFrame(coords=coordinates(st25[7:8]),st25))

spdf26<-as.ppp(SpatialPointsDataFrame(coords=coordinates(st26[7:8]),st26))

spdf27<-as.ppp(SpatialPointsDataFrame(coords=coordinates(st27[7:8]),st27))

spdf28<-as.ppp(SpatialPointsDataFrame(coords=coordinates(st28[7:8]),st28))

spdf29<-as.ppp(SpatialPointsDataFrame(coords=coordinates(st29[7:8]),st29))

spdf30<-as.ppp(SpatialPointsDataFrame(coords=coordinates(st30[7:8]),st30))

spdf31<-as.ppp(SpatialPointsDataFrame(coords=coordinates(st31[7:8]),st31))

spdf32<-as.ppp(SpatialPointsDataFrame(coords=coordinates(st32[7:8]),st32))

spdf33<-as.ppp(SpatialPointsDataFrame(coords=coordinates(st33[7:8]),st33))

spdf34<-as.ppp(SpatialPointsDataFrame(coords=coordinates(st34[7:8]),st34))

spdf35<-as.ppp(SpatialPointsDataFrame(coords=coordinates(st35[7:8]),st35))

spdf36<-as.ppp(SpatialPointsDataFrame(coords=coordinates(st36[7:8]),st36))

spdf37<-as.ppp(SpatialPointsDataFrame(coords=coordinates(st37[7:8]),st37))

spdf38<-as.ppp(SpatialPointsDataFrame(coords=coordinates(st38[7:8]),st38))

spdf39<-as.ppp(SpatialPointsDataFrame(coords=coordinates(st39[7:8]),st39))

spdf40<-as.ppp(SpatialPointsDataFrame(coords=coordinates(st40[7:8]),st40))

spdf41<-as.ppp(SpatialPointsDataFrame(coords=coordinates(st41[7:8]),st41))

spdf42<-as.ppp(SpatialPointsDataFrame(coords=coordinates(st42[7:8]),st42))

spdf43<-as.ppp(SpatialPointsDataFrame(coords=coordinates(st43[7:8]),st43))

spdf44<-as.ppp(SpatialPointsDataFrame(coords=coordinates(st44[7:8]),st44))

spdf45<-as.ppp(SpatialPointsDataFrame(coords=coordinates(st45[7:8]),st45))

spdf46<-as.ppp(SpatialPointsDataFrame(coords=coordinates(st46[7:8]),st46))

spdf47<-as.ppp(SpatialPointsDataFrame(coords=coordinates(st47[7:8]),st47))

spdf48<-as.ppp(SpatialPointsDataFrame(coords=coordinates(st48[7:8]),st48))

spdf49<-as.ppp(SpatialPointsDataFrame(coords=coordinates(st49[7:8]),st49))

spdf50<-as.ppp(SpatialPointsDataFrame(coords=coordinates(st50[7:8]),st50))

spdf51<-as.ppp(SpatialPointsDataFrame(coords=coordinates(st51[7:8]),st51))

spdf52<-as.ppp(SpatialPointsDataFrame(coords=coordinates(st52[7:8]),st52))

spdf53<-as.ppp(SpatialPointsDataFrame(coords=coordinates(st53[7:8]),st53))

spdf54<-as.ppp(SpatialPointsDataFrame(coords=coordinates(st54[7:8]),st54))

spdf55<-as.ppp(SpatialPointsDataFrame(coords=coordinates(st55[7:8]),st55))

spdf56<-as.ppp(SpatialPointsDataFrame(coords=coordinates(st56[7:8]),st56))

spdf57<-as.ppp(SpatialPointsDataFrame(coords=coordinates(st57[7:8]),st57))

spdf58<-as.ppp(SpatialPointsDataFrame(coords=coordinates(st58[7:8]),st58))

spdf59<-as.ppp(SpatialPointsDataFrame(coords=coordinates(st59[7:8]),st59))

spdf60<-as.ppp(SpatialPointsDataFrame(coords=coordinates(st60[7:8]),st60))

spdf61<-as.ppp(SpatialPointsDataFrame(coords=coordinates(st61[7:8]),st61))

kest1<-Kest(spdf1,correction="Ripley")

kest2<-Kest(spdf2,correction="Ripley")

kest3<-Kest(spdf3,correction="Ripley")

kest4<-Kest(spdf4,correction="Ripley")

kest5<-Kest(spdf5,correction="Ripley")

kest6<-Kest(spdf6,correction="Ripley")

kest7<-Kest(spdf7,correction="Ripley")

kest8<-Kest(spdf8,correction="Ripley")

kest9<-Kest(spdf9,correction="Ripley")

kest10<-Kest(spdf10,correction="Ripley")

kest11<-Kest(spdf11,correction="Ripley")

kest12<-Kest(spdf12,correction="Ripley")

kest13<-Kest(spdf13,correction="Ripley")

kest14<-Kest(spdf14,correction="Ripley")

kest15<-Kest(spdf15,correction="Ripley")

kest16<-Kest(spdf16,correction="Ripley")

kest17<-Kest(spdf17,correction="Ripley")

kest18<-Kest(spdf18,correction="Ripley")

kest19<-Kest(spdf19,correction="Ripley")

kest20<-Kest(spdf20,correction="Ripley")

kest21<-Kest(spdf21,correction="Ripley")

kest22<-Kest(spdf22,correction="Ripley")

kest23<-Kest(spdf23,correction="Ripley")

kest24<-Kest(spdf24,correction="Ripley")

kest25<-Kest(spdf25,correction="Ripley")

kest26<-Kest(spdf26,correction="Ripley")

kest27<-Kest(spdf27,correction="Ripley")

kest28<-Kest(spdf28,correction="Ripley")

kest29<-Kest(spdf29,correction="Ripley")

kest30<-Kest(spdf30,correction="Ripley")

kest31<-Kest(spdf31,correction="Ripley")

kest32<-Kest(spdf32,correction="Ripley")

kest33<-Kest(spdf33,correction="Ripley")

kest34<-Kest(spdf34,correction="Ripley")

kest35<-Kest(spdf35,correction="Ripley")

kest36<-Kest(spdf36,correction="Ripley")

kest37<-Kest(spdf37,correction="Ripley")

kest38<-Kest(spdf38,correction="Ripley")

kest39<-Kest(spdf39,correction="Ripley")

kest40<-Kest(spdf40,correction="Ripley")

kest41<-Kest(spdf41,correction="Ripley")

kest42<-Kest(spdf42,correction="Ripley")

kest43<-Kest(spdf43,correction="Ripley")

st1.20m<-mean(kest1$iso-kest1$theo)

st2.20m<-mean(kest2$iso-kest2$theo)

st3.20m<-mean(kest3$iso-kest3$theo)

st4.20m<-mean(kest4$iso-kest4$theo)

st5.20m<-mean(kest5$iso-kest5$theo)

st6.20m<-mean(kest6$iso-kest6$theo)

st7.20m<-mean(kest7$iso-kest7$theo)

st8.20m<-mean(kest8$iso-kest8$theo)

st9.20m<-mean(kest9$iso-kest9$theo)

st10.20m<-mean(kest10$iso-kest10$theo)

st11.20m<-mean(kest11$iso-kest11$theo)

st12.20m<-mean(kest12$iso-kest12$theo)

st13.20m<-mean(kest13$iso-kest13$theo)

st14.20m<-mean(kest14$iso-kest14$theo)

st15.20m<-mean(kest15$iso-kest15$theo)

st16.20m<-mean(kest16$iso-kest16$theo)

st17.20m<-mean(kest17$iso-kest17$theo)

st18.20m<-mean(kest18$iso-kest18$theo)

st19.20m<-mean(kest19$iso-kest19$theo)

st20.20m<-mean(kest20$iso-kest20$theo)

st21.20m<-mean(kest21$iso-kest21$theo)

st22.20m<-mean(kest22$iso-kest22$theo)

st23.20m<-mean(kest23$iso-kest23$theo)

st24.20m<-mean(kest24$iso-kest24$theo)

st25.20m<-mean(kest25$iso-kest25$theo)

st26.20m<-mean(kest26$iso-kest26$theo)

st27.20m<-mean(kest27$iso-kest27$theo)

st28.20m<-mean(kest28$iso-kest28$theo)

st29.20m<-mean(kest29$iso-kest29$theo)

st30.20m<-mean(kest30$iso-kest30$theo)

st31.20m<-mean(kest31$iso-kest31$theo)

st32.20m<-mean(kest32$iso-kest32$theo)

st33.20m<-mean(kest33$iso-kest33$theo)

st34.20m<-mean(kest34$iso-kest34$theo)

st35.20m<-mean(kest35$iso-kest35$theo)

st36.20m<-mean(kest36$iso-kest36$theo)

st37.20m<-mean(kest37$iso-kest37$theo)

st38.20m<-mean(kest38$iso-kest38$theo)

st39.20m<-mean(kest39$iso-kest39$theo)

st40.20m<-mean(kest40$iso-kest40$theo)

st41.20m<-mean(kest41$iso-kest41$theo)

st42.20m<-mean(kest42$iso-kest42$theo)

st43.20m<-mean(kest43$iso-kest43$theo)

st44.20m<-mean(kest44$iso-kest44$theo)

st45.20m<-mean(kest45$iso-kest45$theo)

st46.20m<-mean(kest46$iso-kest46$theo)

st47.20m<-mean(kest47$iso-kest47$theo)

st48.20m<-mean(kest48$iso-kest48$theo)

st49.20m<-mean(kest49$iso-kest49$theo)

st50.20m<-mean(kest50$iso-kest50$theo)

st51.20m<-mean(kest51$iso-kest51$theo)

st52.20m<-mean(kest52$iso-kest52$theo)

st1.20sd<-sd(kest1$iso-kest1$theo)

st2.20sd<-sd(kest2$iso-kest2$theo)

st3.20sd<-sd(kest3$iso-kest3$theo)

st4.20sd<-sd(kest4$iso-kest4$theo)

st5.20sd<-sd(kest5$iso-kest5$theo)

st6.20sd<-sd(kest6$iso-kest6$theo)

st7.20sd<-sd(kest7$iso-kest7$theo)

st8.20sd<-sd(kest8$iso-kest8$theo)

st9.20sd<-sd(kest9$iso-kest9$theo)

st10.20sd<-sd(kest10$iso-kest10$theo)

st11.20sd<-sd(kest11$iso-kest11$theo)

st12.20sd<-sd(kest12$iso-kest12$theo)

st13.20sd<-sd(kest13$iso-kest13$theo)

st14.20sd<-sd(kest14$iso-kest14$theo)

st15.20sd<-sd(kest15$iso-kest15$theo)

st16.20sd<-sd(kest16$iso-kest16$theo)

st17.20sd<-sd(kest17$iso-kest17$theo)

st18.20sd<-sd(kest18$iso-kest18$theo)

st19.20sd<-sd(kest19$iso-kest19$theo)

st20.20sd<-sd(kest20$iso-kest20$theo)

st21.20sd<-sd(kest21$iso-kest21$theo)

st22.20sd<-sd(kest22$iso-kest22$theo)

st23.20sd<-sd(kest23$iso-kest23$theo)

st24.20sd<-sd(kest24$iso-kest24$theo)

st25.20sd<-sd(kest25$iso-kest25$theo)

st26.20sd<-sd(kest26$iso-kest26$theo)

st27.20sd<-sd(kest27$iso-kest27$theo)

st28.20sd<-sd(kest28$iso-kest28$theo)

st29.20sd<-sd(kest29$iso-kest29$theo)

st30.20sd<-sd(kest30$iso-kest30$theo)

st31.20sd<-sd(kest31$iso-kest31$theo)

st32.20sd<-sd(kest32$iso-kest32$theo)

st33.20sd<-sd(kest33$iso-kest33$theo)

st34.20sd<-sd(kest34$iso-kest34$theo)

st35.20sd<-sd(kest35$iso-kest35$theo)

st36.20sd<-sd(kest36$iso-kest36$theo)

st37.20sd<-sd(kest37$iso-kest37$theo)

st38.20sd<-sd(kest38$iso-kest38$theo)

st39.20sd<-sd(kest39$iso-kest39$theo)

st40.20sd<-sd(kest40$iso-kest40$theo)

st41.20sd<-sd(kest41$iso-kest41$theo)

st42.20sd<-sd(kest42$iso-kest42$theo)

st43.20sd<-sd(kest43$iso-kest43$theo)

st44.20sd<-sd(kest44$iso-kest44$theo)

st45.20sd<-sd(kest45$iso-kest45$theo)

st46.20sd<-sd(kest46$iso-kest46$theo)

st47.20sd<-sd(kest47$iso-kest47$theo)

st48.20sd<-sd(kest48$iso-kest48$theo)

st49.20sd<-sd(kest49$iso-kest49$theo)

st50.20sd<-sd(kest50$iso-kest50$theo)

st51.20sd<-sd(kest51$iso-kest51$theo)

st52.20sd<-sd(kest52$iso-kest52$theo)

mean20<-data.frame( mean=rbind(st1.20m,st2.20m,st3.20m,st4.20m,st5.20m,st6.20m,st7.20m,st8.20m,st9.20m,st10.20m, st11.20m,st12.20m,st13.20m,st14.20m,st15.20m,st16.20m,st17.20m,st18.20m,st19.20m,st20.20m, st21.20m,st22.20m,st23.20m,st24.20m,st25.20m,st26.20m,st27.20m,st28.20m,st29.20m,st30.20m, st31.20m,st32.20m,st33.20m,st34.20m,st35.20m,st36.20m,st37.20m,st38.20m,st39.20m,st40.20m), sd=rbind(st1.20sd,st2.20sd,st3.20sd,st4.20sd,st5.20sd,st6.20sd,st7.20sd,st8.20sd,st9.20sd,st10.20sd, st11.20sd,st12.20sd,st13.20sd,st14.20sd,st15.20sd,st16.20sd,st17.20sd,st18.20sd,st19.20sd,st20.20sd, st21.20sd,st22.20sd,st23.20sd,st24.20sd,st25.20sd,st26.20sd,st27.20sd,st28.20sd,st29.20sd,st30.20sd, st31.20sd,st32.20sd,st33.20sd,st34.20sd,st35.20sd,st36.20sd,st37.20sd,st38.20sd,st39.20sd,st40.20sd), depth=(c(1:40)*5)-5,year=c(2020),season=c("2019/20"))

#####-------------2021/22-------------

#### separate each depth strata with detcetd krill swarms

st1<-subset(k22,depth.strata=="0"& NASC>0)

st2<-subset(k22,depth.strata=="5"& NASC>0)

st3<-subset(k22,depth.strata=="10"& NASC>0)

st4<-subset(k22,depth.strata=="15"& NASC>0)

st5<-subset(k22,depth.strata=="20"& NASC>0)

st6<-subset(k22,depth.strata=="25"& NASC>0)

st7<-subset(k22,depth.strata=="30"& NASC>0)

st8<-subset(k22,depth.strata=="35"& NASC>0)

st9<-subset(k22,depth.strata=="40"& NASC>0)

st10<-subset(k22,depth.strata=="45"& NASC>0)

st11<-subset(k22,depth.strata=="50"& NASC>0)

st12<-subset(k22,depth.strata=="55"& NASC>0)

st13<-subset(k22,depth.strata=="60"& NASC>0)

st14<-subset(k22,depth.strata=="65"& NASC>0)

st15<-subset(k22,depth.strata=="70"& NASC>0)

st16<-subset(k22,depth.strata=="75"& NASC>0)

st17<-subset(k22,depth.strata=="80"& NASC>0)

st18<-subset(k22,depth.strata=="85"& NASC>0)

st19<-subset(k22,depth.strata=="90"& NASC>0)

st20<-subset(k22,depth.strata=="95"& NASC>0)

st21<-subset(k22,depth.strata=="100"& NASC>0)

st22<-subset(k22,depth.strata=="105"& NASC>0)

st23<-subset(k22,depth.strata=="110"& NASC>0)

st24<-subset(k22,depth.strata=="115"& NASC>0)

st25<-subset(k22,depth.strata=="120"& NASC>0)

st26<-subset(k22,depth.strata=="125"& NASC>0)

st27<-subset(k22,depth.strata=="130"& NASC>0)

st28<-subset(k22,depth.strata=="135"& NASC>0)

st29<-subset(k22,depth.strata=="140"& NASC>0)

st30<-subset(k22,depth.strata=="145"& NASC>0)

st31<-subset(k22,depth.strata=="150"& NASC>0)

st32<-subset(k22,depth.strata=="155"& NASC>0)

st33<-subset(k22,depth.strata=="160"& NASC>0)

st34<-subset(k22,depth.strata=="165"& NASC>0)

st35<-subset(k22,depth.strata=="170"& NASC>0)

st36<-subset(k22,depth.strata=="175"& NASC>0)

st37<-subset(k22,depth.strata=="180"& NASC>0)

st38<-subset(k22,depth.strata=="185"& NASC>0)

st39<-subset(k22,depth.strata=="190"& NASC>0)

st40<-subset(k22,depth.strata=="195"& NASC>0)

st41<-subset(k22,depth.strata=="200"& NASC>0)

st42<-subset(k22,depth.strata=="205"& NASC>0)

st43<-subset(k22,depth.strata=="210"& NASC>0)

st44<-subset(k22,depth.strata=="215"& NASC>0)

st45<-subset(k22,depth.strata=="220"& NASC>0)

st46<-subset(k22,depth.strata=="225"& NASC>0)

st47<-subset(k22,depth.strata=="230"& NASC>0)

st48<-subset(k22,depth.strata=="235"& NASC>0)

st49<-subset(k22,depth.strata=="240"& NASC>0)

st50<-subset(k22,depth.strata=="245"& NASC>0)

st51<-subset(k22,depth.strata=="250"& NASC>0)

st52<-subset(k22,depth.strata=="255"& NASC>0)

st53<-subset(k22,depth.strata=="260"& NASC>0)

st54<-subset(k22,depth.strata=="265"& NASC>0)

st55<-subset(k22,depth.strata=="270"& NASC>0)

st56<-subset(k22,depth.strata=="275"& NASC>0)

st57<-subset(k22,depth.strata=="280"& NASC>0)

st58<-subset(k22,depth.strata=="285"& NASC>0)

st59<-subset(k22,depth.strata=="290"& NASC>0)

st60<-subset(k22,depth.strata=="295"& NASC>0)

st61<-subset(k22,depth.strata=="300"& NASC>0)

### create a spatial points data frame for each

spdf1<-as.ppp(SpatialPointsDataFrame(coords=coordinates(st1[7:8]),st1))

spdf2<-as.ppp(SpatialPointsDataFrame(coords=coordinates(st2[7:8]),st2))

spdf3<-as.ppp(SpatialPointsDataFrame(coords=coordinates(st3[7:8]),st3))

spdf4<-as.ppp(SpatialPointsDataFrame(coords=coordinates(st4[7:8]),st4))

spdf5<-as.ppp(SpatialPointsDataFrame(coords=coordinates(st5[7:8]),st5))

spdf6<-as.ppp(SpatialPointsDataFrame(coords=coordinates(st6[7:8]),st6))

spdf7<-as.ppp(SpatialPointsDataFrame(coords=coordinates(st7[7:8]),st7))

spdf8<-as.ppp(SpatialPointsDataFrame(coords=coordinates(st8[7:8]),st8))

spdf9<-as.ppp(SpatialPointsDataFrame(coords=coordinates(st9[7:8]),st9))

spdf10<-as.ppp(SpatialPointsDataFrame(coords=coordinates(st10[7:8]),st10))

spdf11<-as.ppp(SpatialPointsDataFrame(coords=coordinates(st11[7:8]),st11))

spdf12<-as.ppp(SpatialPointsDataFrame(coords=coordinates(st12[7:8]),st12))

spdf13<-as.ppp(SpatialPointsDataFrame(coords=coordinates(st13[7:8]),st13))

spdf14<-as.ppp(SpatialPointsDataFrame(coords=coordinates(st14[7:8]),st14))

spdf15<-as.ppp(SpatialPointsDataFrame(coords=coordinates(st15[7:8]),st15))

spdf16<-as.ppp(SpatialPointsDataFrame(coords=coordinates(st16[7:8]),st16))

spdf17<-as.ppp(SpatialPointsDataFrame(coords=coordinates(st17[7:8]),st17))

spdf18<-as.ppp(SpatialPointsDataFrame(coords=coordinates(st18[7:8]),st18))

spdf19<-as.ppp(SpatialPointsDataFrame(coords=coordinates(st19[7:8]),st19))

spdf20<-as.ppp(SpatialPointsDataFrame(coords=coordinates(st20[7:8]),st20))

spdf21<-as.ppp(SpatialPointsDataFrame(coords=coordinates(st21[7:8]),st21))

spdf22<-as.ppp(SpatialPointsDataFrame(coords=coordinates(st22[7:8]),st22))

spdf23<-as.ppp(SpatialPointsDataFrame(coords=coordinates(st23[7:8]),st23))

spdf24<-as.ppp(SpatialPointsDataFrame(coords=coordinates(st24[7:8]),st24))

spdf25<-as.ppp(SpatialPointsDataFrame(coords=coordinates(st25[7:8]),st25))

spdf26<-as.ppp(SpatialPointsDataFrame(coords=coordinates(st26[7:8]),st26))

spdf27<-as.ppp(SpatialPointsDataFrame(coords=coordinates(st27[7:8]),st27))

spdf28<-as.ppp(SpatialPointsDataFrame(coords=coordinates(st28[7:8]),st28))

spdf29<-as.ppp(SpatialPointsDataFrame(coords=coordinates(st29[7:8]),st29))

spdf30<-as.ppp(SpatialPointsDataFrame(coords=coordinates(st30[7:8]),st30))

spdf31<-as.ppp(SpatialPointsDataFrame(coords=coordinates(st31[7:8]),st31))

spdf32<-as.ppp(SpatialPointsDataFrame(coords=coordinates(st32[7:8]),st32))

spdf33<-as.ppp(SpatialPointsDataFrame(coords=coordinates(st33[7:8]),st33))

spdf34<-as.ppp(SpatialPointsDataFrame(coords=coordinates(st34[7:8]),st34))

spdf35<-as.ppp(SpatialPointsDataFrame(coords=coordinates(st35[7:8]),st35))

spdf36<-as.ppp(SpatialPointsDataFrame(coords=coordinates(st36[7:8]),st36))

spdf37<-as.ppp(SpatialPointsDataFrame(coords=coordinates(st37[7:8]),st37))

spdf38<-as.ppp(SpatialPointsDataFrame(coords=coordinates(st38[7:8]),st38))

spdf39<-as.ppp(SpatialPointsDataFrame(coords=coordinates(st39[7:8]),st39))

spdf40<-as.ppp(SpatialPointsDataFrame(coords=coordinates(st40[7:8]),st40))

spdf41<-as.ppp(SpatialPointsDataFrame(coords=coordinates(st41[7:8]),st41))

spdf42<-as.ppp(SpatialPointsDataFrame(coords=coordinates(st42[7:8]),st42))

spdf43<-as.ppp(SpatialPointsDataFrame(coords=coordinates(st43[7:8]),st43))

spdf44<-as.ppp(SpatialPointsDataFrame(coords=coordinates(st44[7:8]),st44))

spdf45<-as.ppp(SpatialPointsDataFrame(coords=coordinates(st45[7:8]),st45))

spdf46<-as.ppp(SpatialPointsDataFrame(coords=coordinates(st46[7:8]),st46))

spdf47<-as.ppp(SpatialPointsDataFrame(coords=coordinates(st47[7:8]),st47))

spdf48<-as.ppp(SpatialPointsDataFrame(coords=coordinates(st48[7:8]),st48))

spdf49<-as.ppp(SpatialPointsDataFrame(coords=coordinates(st49[7:8]),st49))

spdf50<-as.ppp(SpatialPointsDataFrame(coords=coordinates(st50[7:8]),st50))

spdf51<-as.ppp(SpatialPointsDataFrame(coords=coordinates(st51[7:8]),st51))

spdf52<-as.ppp(SpatialPointsDataFrame(coords=coordinates(st52[7:8]),st52))

spdf53<-as.ppp(SpatialPointsDataFrame(coords=coordinates(st53[7:8]),st53))

spdf54<-as.ppp(SpatialPointsDataFrame(coords=coordinates(st54[7:8]),st54))

spdf55<-as.ppp(SpatialPointsDataFrame(coords=coordinates(st55[7:8]),st55))

spdf56<-as.ppp(SpatialPointsDataFrame(coords=coordinates(st56[7:8]),st56))

spdf57<-as.ppp(SpatialPointsDataFrame(coords=coordinates(st57[7:8]),st57))

spdf58<-as.ppp(SpatialPointsDataFrame(coords=coordinates(st58[7:8]),st58))

spdf59<-as.ppp(SpatialPointsDataFrame(coords=coordinates(st59[7:8]),st59))

spdf60<-as.ppp(SpatialPointsDataFrame(coords=coordinates(st60[7:8]),st60))

spdf61<-as.ppp(SpatialPointsDataFrame(coords=coordinates(st61[7:8]),st61))

kest1<-Kest(spdf1,correction="Ripley")

kest2<-Kest(spdf2,correction="Ripley")

kest3<-Kest(spdf3,correction="Ripley")

kest4<-Kest(spdf4,correction="Ripley")

kest5<-Kest(spdf5,correction="Ripley")

kest6<-Kest(spdf6,correction="Ripley")

kest7<-Kest(spdf7,correction="Ripley")

kest8<-Kest(spdf8,correction="Ripley")

kest9<-Kest(spdf9,correction="Ripley")

kest10<-Kest(spdf10,correction="Ripley")

kest11<-Kest(spdf11,correction="Ripley")

kest12<-Kest(spdf12,correction="Ripley")

kest13<-Kest(spdf13,correction="Ripley")

kest14<-Kest(spdf14,correction="Ripley")

kest15<-Kest(spdf15,correction="Ripley")

kest16<-Kest(spdf16,correction="Ripley")

kest17<-Kest(spdf17,correction="Ripley")

kest18<-Kest(spdf18,correction="Ripley")

kest19<-Kest(spdf19,correction="Ripley")

kest20<-Kest(spdf20,correction="Ripley")

kest21<-Kest(spdf21,correction="Ripley")

kest22<-Kest(spdf22,correction="Ripley")

kest23<-Kest(spdf23,correction="Ripley")

kest24<-Kest(spdf24,correction="Ripley")

kest25<-Kest(spdf25,correction="Ripley")

kest26<-Kest(spdf26,correction="Ripley")

kest27<-Kest(spdf27,correction="Ripley")

kest28<-Kest(spdf28,correction="Ripley")

kest29<-Kest(spdf29,correction="Ripley")

kest30<-Kest(spdf30,correction="Ripley")

kest31<-Kest(spdf31,correction="Ripley")

kest32<-Kest(spdf32,correction="Ripley")

kest33<-Kest(spdf33,correction="Ripley")

kest34<-Kest(spdf34,correction="Ripley")

kest35<-Kest(spdf35,correction="Ripley")

kest36<-Kest(spdf36,correction="Ripley")

kest37<-Kest(spdf37,correction="Ripley")

kest38<-Kest(spdf38,correction="Ripley")

kest39<-Kest(spdf39,correction="Ripley")

kest40<-Kest(spdf40,correction="Ripley")

kest41<-Kest(spdf41,correction="Ripley")

kest42<-Kest(spdf42,correction="Ripley")

kest43<-Kest(spdf43,correction="Ripley")

st1.22m<-mean(kest1$iso-kest1$theo)

st2.22m<-mean(kest2$iso-kest2$theo)

st3.22m<-mean(kest3$iso-kest3$theo)

st4.22m<-mean(kest4$iso-kest4$theo)

st5.22m<-mean(kest5$iso-kest5$theo)

st6.22m<-mean(kest6$iso-kest6$theo)

st7.22m<-mean(kest7$iso-kest7$theo)

st8.22m<-mean(kest8$iso-kest8$theo)

st9.22m<-mean(kest9$iso-kest9$theo)

st10.22m<-mean(kest10$iso-kest10$theo)

st11.22m<-mean(kest11$iso-kest11$theo)

st12.22m<-mean(kest12$iso-kest12$theo)

st13.22m<-mean(kest13$iso-kest13$theo)

st14.22m<-mean(kest14$iso-kest14$theo)

st15.22m<-mean(kest15$iso-kest15$theo)

st16.22m<-mean(kest16$iso-kest16$theo)

st17.22m<-mean(kest17$iso-kest17$theo)

st18.22m<-mean(kest18$iso-kest18$theo)

st19.22m<-mean(kest19$iso-kest19$theo)

st20.22m<-mean(kest20$iso-kest20$theo)

st21.22m<-mean(kest21$iso-kest21$theo)

st22.22m<-mean(kest22$iso-kest22$theo)

st23.22m<-mean(kest23$iso-kest23$theo)

st24.22m<-mean(kest24$iso-kest24$theo)

st25.22m<-mean(kest25$iso-kest25$theo)

st26.22m<-mean(kest26$iso-kest26$theo)

st27.22m<-mean(kest27$iso-kest27$theo)

st28.22m<-mean(kest28$iso-kest28$theo)

st29.22m<-mean(kest29$iso-kest29$theo)

st30.22m<-mean(kest30$iso-kest30$theo)

st31.22m<-mean(kest31$iso-kest31$theo)

st32.22m<-mean(kest32$iso-kest32$theo)

st33.22m<-mean(kest33$iso-kest33$theo)

st34.22m<-mean(kest34$iso-kest34$theo)

st35.22m<-mean(kest35$iso-kest35$theo)

st36.22m<-mean(kest36$iso-kest36$theo)

st37.22m<-mean(kest37$iso-kest37$theo)

st38.22m<-mean(kest38$iso-kest38$theo)

st39.22m<-mean(kest39$iso-kest39$theo)

st40.22m<-mean(kest40$iso-kest40$theo)

st41.22m<-mean(kest41$iso-kest41$theo)

st42.22m<-mean(kest42$iso-kest42$theo)

st43.22m<-mean(kest43$iso-kest43$theo)

st44.22m<-mean(kest44$iso-kest44$theo)

st45.22m<-mean(kest45$iso-kest45$theo)

st46.22m<-mean(kest46$iso-kest46$theo)

st47.22m<-mean(kest47$iso-kest47$theo)

st48.22m<-mean(kest48$iso-kest48$theo)

st49.22m<-mean(kest49$iso-kest49$theo)

st50.22m<-mean(kest50$iso-kest50$theo)

st51.22m<-mean(kest51$iso-kest51$theo)

st52.22m<-mean(kest52$iso-kest52$theo)

st1.22sd<-sd(kest1$iso-kest1$theo)

st2.22sd<-sd(kest2$iso-kest2$theo)

st3.22sd<-sd(kest3$iso-kest3$theo)

st4.22sd<-sd(kest4$iso-kest4$theo)

st5.22sd<-sd(kest5$iso-kest5$theo)

st6.22sd<-sd(kest6$iso-kest6$theo)

st7.22sd<-sd(kest7$iso-kest7$theo)

st8.22sd<-sd(kest8$iso-kest8$theo)

st9.22sd<-sd(kest9$iso-kest9$theo)

st10.22sd<-sd(kest10$iso-kest10$theo)

st11.22sd<-sd(kest11$iso-kest11$theo)

st12.22sd<-sd(kest12$iso-kest12$theo)

st13.22sd<-sd(kest13$iso-kest13$theo)

st14.22sd<-sd(kest14$iso-kest14$theo)

st15.22sd<-sd(kest15$iso-kest15$theo)

st16.22sd<-sd(kest16$iso-kest16$theo)

st17.22sd<-sd(kest17$iso-kest17$theo)

st18.22sd<-sd(kest18$iso-kest18$theo)

st19.22sd<-sd(kest19$iso-kest19$theo)

st20.22sd<-sd(kest20$iso-kest20$theo)

st21.22sd<-sd(kest21$iso-kest21$theo)

st22.22sd<-sd(kest22$iso-kest22$theo)

st23.22sd<-sd(kest23$iso-kest23$theo)

st24.22sd<-sd(kest24$iso-kest24$theo)

st25.22sd<-sd(kest25$iso-kest25$theo)

st26.22sd<-sd(kest26$iso-kest26$theo)

st27.22sd<-sd(kest27$iso-kest27$theo)

st28.22sd<-sd(kest28$iso-kest28$theo)

st29.22sd<-sd(kest29$iso-kest29$theo)

st30.22sd<-sd(kest30$iso-kest30$theo)

st31.22sd<-sd(kest31$iso-kest31$theo)

st32.22sd<-sd(kest32$iso-kest32$theo)

st33.22sd<-sd(kest33$iso-kest33$theo)

st34.22sd<-sd(kest34$iso-kest34$theo)

st35.22sd<-sd(kest35$iso-kest35$theo)

st36.22sd<-sd(kest36$iso-kest36$theo)

st37.22sd<-sd(kest37$iso-kest37$theo)

st38.22sd<-sd(kest38$iso-kest38$theo)

st39.22sd<-sd(kest39$iso-kest39$theo)

st40.22sd<-sd(kest40$iso-kest40$theo)

st41.22sd<-sd(kest41$iso-kest41$theo)

st42.22sd<-sd(kest42$iso-kest42$theo)

st43.22sd<-sd(kest43$iso-kest43$theo)

st44.22sd<-sd(kest44$iso-kest44$theo)

st45.22sd<-sd(kest45$iso-kest45$theo)

st46.22sd<-sd(kest46$iso-kest46$theo)

st47.22sd<-sd(kest47$iso-kest47$theo)

st48.22sd<-sd(kest48$iso-kest48$theo)

st49.22sd<-sd(kest49$iso-kest49$theo)

st50.22sd<-sd(kest50$iso-kest50$theo)

st51.22sd<-sd(kest51$iso-kest51$theo)

st52.22sd<-sd(kest52$iso-kest52$theo)

mean22<-data.frame( mean=rbind(st1.22m,st2.22m,st3.22m,st4.22m,st5.22m,st6.22m,st7.22m,st8.22m,st9.22m,st10.22m, st11.22m,st12.22m,st13.22m,st14.22m,st15.22m,st16.22m,st17.22m,st18.22m,st19.22m,st20.22m, st21.22m,st22.22m,st23.22m,st24.22m,st25.22m,st26.22m,st27.22m,st28.22m,st29.22m,st30.22m, st31.22m,st32.22m,st33.22m,st34.22m,st35.22m,st36.22m,st37.22m,st38.22m,st39.22m,st40.22m), sd=rbind(st1.22sd,st2.22sd,st3.22sd,st4.22sd,st5.22sd,st6.22sd,st7.22sd,st8.22sd,st9.22sd,st10.22sd, st11.22sd,st12.22sd,st13.22sd,st14.22sd,st15.22sd,st16.22sd,st17.22sd,st18.22sd,st19.22sd,st20.22sd, st21.22sd,st22.22sd,st23.22sd,st24.22sd,st25.22sd,st26.22sd,st27.22sd,st28.22sd,st29.22sd,st30.22sd, st31.22sd,st32.22sd,st33.22sd,st34.22sd,st35.22sd,st36.22sd,st37.22sd,st38.22sd,st39.22sd,st40.22sd), depth=(c(1:40)*5)-5,year=c(2022),season=c("2021/22"))

agg<-rbind(mean19,mean20,mean22)

plot(kest2, sqrt(./pi) ~ r, ylab="L(r)", main="L function for cells")

plot(kest10, sqrt(./pi) ~ r, ylab="L(r)", main="L function for cells")

plot(kest20, sqrt(./pi) ~ r, ylab="L(r)", main="L function for cells")

###----------permanova aggregation ---------------

library(PERMANOVA)

head(agg)

Xtdag=IniTransform(as.matrix(agg$mean),

transform="Standardize columns")

TDag=DistContinuous(Xtdag)

ftdag<-PERMANOVA(TDag,group=as.factor(agg$season),nperm=999)

print(ftdag)$Initial$Global

summary(ftdag)

#### -----------plots---------------------

head(agg)

aggm<-ddply(agg, c("season","depth"), summarise,

mean=mean(mean),

sd=mean(sd))

head(chdf)

ggplot((kallm), aes(-1*depth.strata,mean,colour=season,linetype=season,shape=season))+

geom_vline(xintercept=-87,colour="red",

linetype="dashed")+

geom_vline(xintercept=-50,colour="blue",

linetype="dotted")+

stat_smooth(span=0.25,se=F)+

geom_errorbar(aes(ymin=mean-se,ymax=mean+se))+

geom_point()+

ylab("Nautical area scattering coefficient")+xlab("Depth (m)")+

coord_flip()+theme_bw()+th+xlim(-200,0)+

scale_colour_manual(values=c("blue","red"))+

scale_linetype_manual(values=c("dashed","dotted"))+

ggtitle(label="a. Krill biomass")+

ggplot((aggm), aes(-1*depth,mean,colour=season,linetype=season,shape=season))+

geom_hline(yintercept=0,colour="grey50",linetype="dotted")+

geom_vline(xintercept=-87,colour="red",

linetype="dashed")+

geom_vline(xintercept=-50,colour="blue",

linetype="dotted")+

stat_smooth(span=0.25,se=F)+

geom_errorbar(aes(ymin=mean-sd,ymax=mean+sd))+

geom_point()+

ylab("Ripley's K")+xlab("Depth (m)")+

coord_flip()+theme_bw()+th+xlim(-200,0)+

scale_colour_manual(values=c("blue","red"))+

scale_linetype_manual(values=c("dashed","dotted"))+

ggtitle(label="b. Krill swarm aggregation")+

ggplot((chdf), aes(-1*BDepM,WigS,colour=Season2,linetype=Season2,shape=Season2))+

geom_vline(xintercept=-87,colour="red",

linetype="dashed")+

geom_vline(xintercept=-50,colour="blue",

linetype="dotted")+

stat_smooth(span=0.65,se=F)+

geom_point(alpha=0.25)+

ylab("Cumulative number of wiggles")+xlab("Mean diving depth (m)")+

coord_flip()+theme_bw()+th+xlim(-200,0)+

scale_colour_manual(values=c("blue","red"))+

scale_linetype_manual(values=c("dashed","dotted"))+

ggtitle(label="c. Penguin foragin effort")

### ------------This is the end!----------------------
